# PepT1 Expression Helps Maintain Intestinal Homeostasis by Mediating the Differential Expression of miRNAs along the Crypt-Villus Axis

**DOI:** 10.1038/srep27119

**Published:** 2016-06-01

**Authors:** Yuchen Zhang, Emilie Viennois, Mingzhen Zhang, Bo Xiao, Moon Kwon Han, Lewins Walter, Pallavi Garg, Didier Merlin

**Affiliations:** 1 Institute for Biomedical Sciences, Center for Diagnostics and Therapeutics, Georgia State University, Atlanta, Georgia, 30302, USA; 2 Institute for Clean Energy and Advanced Materials, Faculty for Materials and Energy, Southwest University, Chongqing, 400715, P. R. China; 3 Department of Biology, Georgia State University, Atlanta, Georgia, 30302, USA; 4 Atlanta Veterans Affairs Medical Center, Decatur, Georgia, 30033, USA

## Abstract

In the jejunum, PepT1 is particularly enriched in the well-differentiated absorptive epithelial cells in the villi. Studies of expression and function of PepT1 along the crypt-villus axis demonstrated that this protein is crucial to the process of di/tripeptide absorption. We recently exhibited that PepT1 plays an important role in multiple biological functions, including the ability to regulate the expression/secretion of specific microRNAs (miRNAs) and the expression levels of multiple proteins. In this study, we observed that PepT1 knockout (KO) mice exhibited reduced body weight and shorten intestinal microvilli. We then examined the expression levels of various miRNAs and their target proteins along the crypt-villi axis in the jejunum of PepT1 KO mice. We found that PepT1 KO altered the distribution of miRNAs along the crypt-villus axis and changed the miRNA profiles of both villi and crypts. Using miRNA-target prediction and 2D-DIGE/mass spectrometry on villi and crypts samples, we found that ablation of PepT1 further directly or indirectly altered expression levels of certain protein targets. Collectively, our results suggest that PepT1 contributes to maintain balance of homeostasis and proper functions in the small intestine, and dysregulated miRNAs and proteins along the crypt-villus axis are highly related to this process.

One of the most significant functions of small intestine is the absorption of the nutrients including di/tri-peptides from the diet. This process is mediated by peptide transport activity^1^,^2^. The trans-membrane protein, *intestinal H*^+^ *-coupled oligo-peptide transporter* (PepT1), which is a member of the SLC15 family of proton-oligopeptide cotransporters, has been found to mediate this activity. PepT1 transports di/tri-peptides (but not free amino acids or peptides with more than three amino residues) from the intestinal lumen into epithelial cells^3^,^4^. Moreover, it was recently shown that PepT1 also transport anti-inflammatory tri-peptides (*e.g.*, KPV and VPY^5^,^6^), peptidomimetic drugs (*e.g.*, β-lactam antibiotics, antiviral drugs and antineoplastic agents^7^,^8^,^9^,^10^), and bacterial proinflammatory peptides [*e.g.*, muramyl dipeptide (MDP)^11^, formyl-methionyl-leucyl-phenylalanine (fMLP)^12^, and *L*-Ala-γ-D-Glu-meso-DAP (Tri-DAP)^13^]. The absorption of bacterial oligopeptides was found to further trigger NF-κB signaling and induce inflammation^13^,^14^,^15^. These results indicate that PepT1, through its transporter activity, plays an important role in the intestine under physiological and pathological conditions, and this protein may thus be a candidate target for therapeutic applications.

It has been reported that PepT1 is mostly expressed in the small intestine, but in limited level or absent in the colon, kidney, pancreas and bile duct^16^,^17^,^18^,^19^,^20^. However, the expression of colonic PepT1 is known to be induced under conditions of chronic inflammation, such as inflammatory bowel disease (IBD)^21^,^22^. Our group and others have also demonstrated that PepT1 is expressed in immune cells, such as macrophages, that are closely related to the lamina propria of the small and large intestines^23^,^24^,^25^.

In the small intestine, PepT1 is mostly expressed in the ileum and jejunum, associated with lipid rafts^17^,^26^. In the jejunum, PepT1 is particularly enriched in the villi, but poorly or not expressed in the goblet cells and less-differentiated epithelial cells of the crypts^17^,^26^. The expression/function of PepT1 along the crypt-villus axis suggests that the protein is specific to the well differentiated absorptive epithelial cells, strengthening the notion that PepT1 is important to the absorption process^17^,^26^.

We recently demonstrated that PepT1 plays important roles in multiple biological functions in the small intestine. The expression of PepT1 regulates the expression/secretion of specific microRNAs (miRNAs), to thereby regulate the expression levels of multiple proteins^27^. Intestinal miRNA expression is essential for maintaining intestinal homeostasis. In the intestinal epithelium, conditional ablation of Dicer1, which is obligatory for miRNA processing, was reported to trigger increased inflammation and disorganization of the epithelium^28^,^29^. Hence, it is crucial to investigate the effect of PepT1 expression on the miRNA expression and the crosstalk between these miRNAs and their target proteins.

Maintaining the intestinal homeostasis is crucial for a physiological equilibrium. A breach on that equilibrium can facilitate development of intestinal diseases such as inflammatory bowel disease. To get a better understanding of PepT1 functions and their influence on the small intestinal homeostasis, we first investigated miRNA expression patterns along the crypt/villus axis in the jejunum of wild-type (WT) mice, where PepT1 is highly expressed. We then examined the expression levels of various miRNAs and their potential target proteins along the crypt/villus axis in the jejunum of PepT1 KO mice, in an effort to identify PepT1-dependent miRNAs in this tissue. Our results support the emerging idea that PepT1 plays an important role in multiple biological processes, as we show for the first time that it critically regulates directly or indirectly the miRNA and protein expression along the crypt/villus axis in the jejunum, and thus helps maintain intestinal homeostasis.

## Results

### PepT1 knockout reduces body weight and size of intestinal microvilli

**Figure 1:**
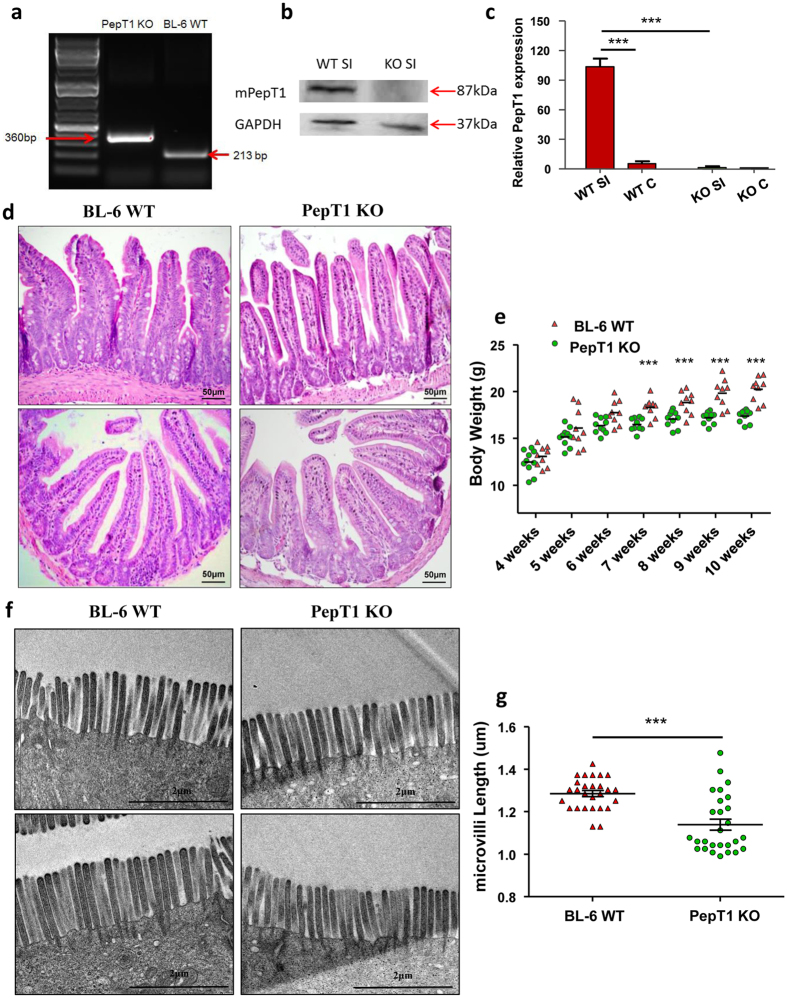
PepT1 knockout reduces mouse body weight and the size of intestinal microvilli. (**a**) Genotyping was performed on PepT1 KO and BL-6 WT mice. Homozygous PepT1 KO mice and appropriate BL-6 WT controls were used in our studies. (**b**,**c**) PepT1 expression levels in the small intestines of PepT1 KO and BL-6 WT mice were detected by Western blotting (**b**) and qRT-PCR (**c**). (**d**) Representative H&E-stained sections of small intestines from BL-6 WT and PepT1 KO mice showed no significant gross morphology difference. Original magnification, 20×. Scale bars, 50 μm. (**e**) Body weights measured at the indicated ages were significantly higher in BL-6 WT mice than in PepT1 KO mice (PepT1 KO: n = 9/group, BL-6 WT: n = 9/group ***P < 0.001). (***f***,**g**) Transmission electron photomicrographs showed abnormal morphology and smaller microvilli on the apical membrane of jejunal enterocytes from PepT1 KO mice compared to those of BL-6 WT controls. Scale bars, 2 μm; n = 27 microvilli/group; and *P < 0.05, ***P < 0.001.

To study the role of PepT1 in small intestinal homeostasis toward the crypt-villus axis during intestinal epithelial cell differentiation, we obtained PepT1 KO mice from Deltagen (San Mateo, CA). Homozygous PepT1 KO mice were identified using a multiplex RT-PCR strategy (Fig. 1a). Mice verified as homozygous and their WT controls were used for further study. Western blotting and qRT-PCR indicated that PepT1 protein and mRNA, respectively, were not expressed in the small intestines of PepT1 KO mice, but were expressed in their WT controls (Fig. 1b,c). PepT1 expression was much lower in the colon than in the small intestine of WT mice, even with this residual PepT1 absent from colon tissue of PepT1 KO mice (Fig. 1c). Histologic examination of small intestine sections showed no obvious differences between PepT1 KO and WT mice (Fig. 1d). However, the body weight of PepT1 KO mice (n = 9) was significantly lower than that of WT animals (n = 9) at week 7 (16.48 ± 0.70 vs 18.30 ± 1.03 g), week 8 (17.06 ± 0.87 vs. 18.82 ± 1.22 g), week 9 (17.19 ± 0.63 vs. 19.82 ± 1.50 g), and week 10 (17.38 ± 0.07 vs. 20.23 ± 1.31 g) (Fig. 1e). Furthermore, examination of ultrastructure by transmission electron microscopy showed abnormal morphology in PepT1 KO mice. The size of microvilli at the apical membranes of enterocytes was much lower in PepT1 KO than in WT animals (1.14 ± 0.14 vs. 1.29 ± 0.07 mm; n = 27) (Fig. 1f,g), suggesting that knockout of PepT1 in the small intestine resulted in defective microvilli at the brush borders of apical surfaces of mouse villus enterocytes. Because microvilli greatly increase the surface area of the small intestine for digestion/absorption, these data suggest that intestinal absorption is decreased in PepT1 KO compared with WT mice.

### Isolation of villi and crypt epithelial cells of the jejunum from WT and PepT1 KO mice

**Figure 2:**
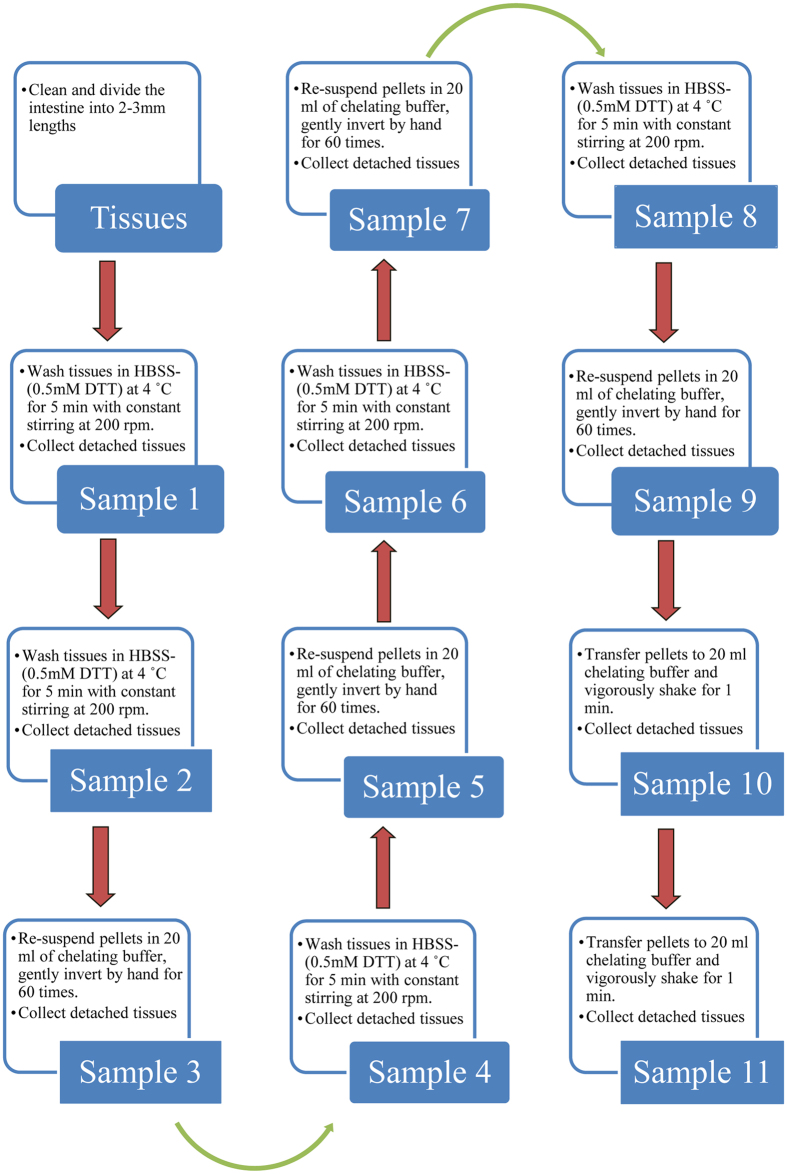
Flow diagram of the low-temperature method used to obtain villi and crypts from intestinal epithelial cells at 4 °C. Epithelia of the jejunum were isolated from crypts and villi of the small intestines of 8-week-old BL-6 WT and PepT1 KO female mice using the previously described low-temperature method^30^.

**Figure 3:**
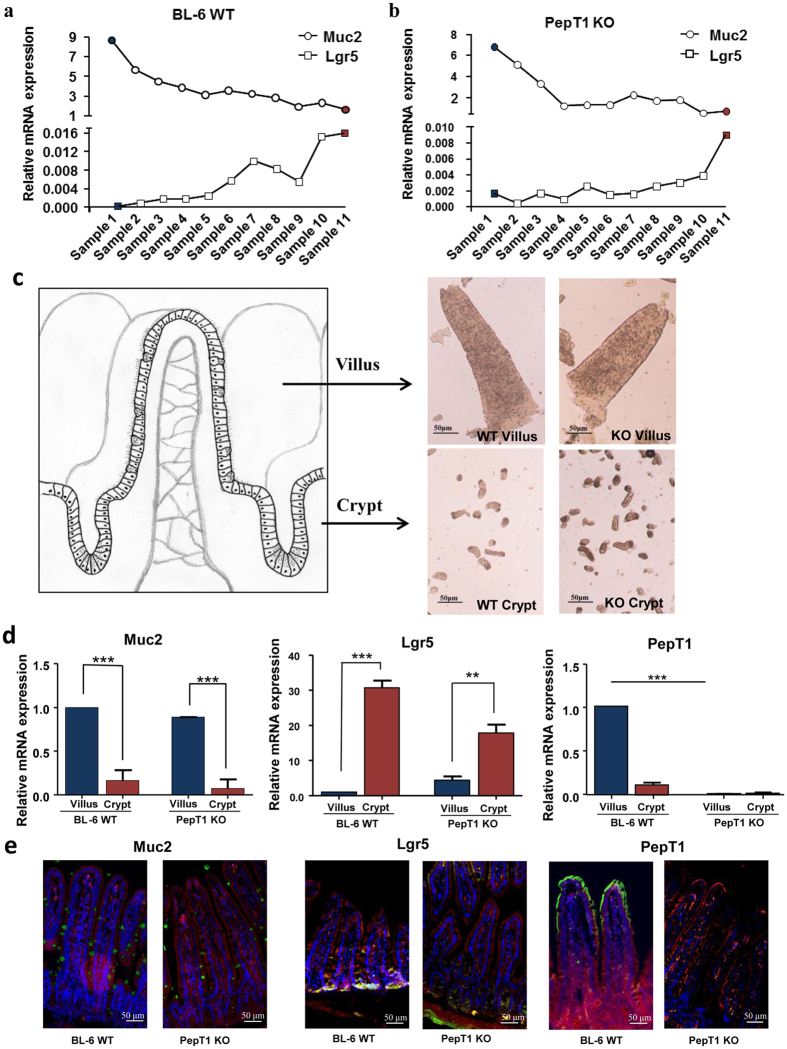
Isolation of villi and crypt epithelial cells of the jejunum from BL-6 WT and PepT1 KO mice. (**a**) Total RNAs were extracted from the different fractions collected from BL-6 WT mice using the low-temperature method, and the expression levels of Muc2 (as a villus marker) and Lgr5 (as a crypt marker) were assessed by qRT-PCR. (**b**) Total RNAs were extracted from different fractions collected from PepT1 KO mice, and the expression levels of Muc2 (as a villus marker) and Lgr5 (as a crypt marker) were assessed by qRT-PCR. (**c**) Pictures of selected villi and crypts fractions were taken with a Nikon Eclipse TS100 microscope at 10× magnifications. Scale bar, 50 μm. (**d**) Total RNAs were extracted from selected villus and crypt fractions from BL-6 WT and PepT1 KO mice. The expression levels of Muc2, Lgr5 and mPepT1 in the villi (blue bars) and crypts (magenta) of BL-6 WT and PepT1 KO mice were further assessed by qRT-PCR (n = 5; and **P < 0.005, ***P < 0.001). (**e**) The expression of Muc2, Lgr5 and mPepT1 in the tissue were confirmed by immunofluorescence. Muc2, Lgr5, mPepT1 were immunostained using anti-Muc2, anti-Lgr5 and anti-PepT1 (FITC, green), respectively, F-actin was stained using phalloidin (TRITC, red), and cell nuclei were stained using DAPI (blue). Separate pictures were taken at 20× for each filter, and the images were merged. Scale bar, 50 μm.

Morphological alteration (shorten microvilli) in PepT1 KO mice has been described. To further evaluate if that change is associated to molecular alterations along the crypt-villus axis, we isolated villi and crypts from the jejunum of 8 week-old PepT1 KO mice and their WT controls using a low-temperature method^30^, as described in the methods (Fig. 2). Total RNA from sample 1 to sample 11 were isolated. qRT-PCR analysis showed that the levels of villus markers including PepT1 mRNA in WT animals and mucin 2 mRNA (Muc2) in both WT and PepT1 KO animals were enriched in sample 1 (Fig. 3a,b and Supplementary Fig. 1) but were less enriched in sample 11 (Fig. 3a,b and Supplementary Fig. 1). Conversely, the levels of mRNA encoding leucine-rich repeat containing protein-coupled receptor 5 (Lgr5), a stem cell marker located at the bottom of the crypts, were enriched in sample 11, but less enriched in sample 1 in both WT and PepT1 KO mice (Fig. 3a,b). As PepT1 and Muc2 were shown to be markers of well differentiated intestinal epithelial cells^31^,^32^, and as Lgr5 is a marker of crypt stem cells^33^, these results suggest that sample 1 is the purest villus isolate whereas sample 11 is the purest crypt isolate. Figure 3c shows the visual appearance of the extracted villus and crypts fractions isolated from the jejunum of WT and PepT1 KO mice. Further qRT-PCR of the isolated samples confirmed that isolated villi from WT mice express high levels of PepT1 and Muc2 and low levels of Lgr5 when compared with isolated crypts (Fig. 3d). The isolated villi from PepT1 KO mice exhibited higher levels of Muc2 expression and lower levels of Lgr5 expression when compared with isolated crypts, with neither villi nor crypts expressing PepT1 (Fig. 3d). These results indicate that villi and crypts can be isolated from WT and PepT1 KO mice with high purity and quality. The differential expression of Muc2, Lgr5 and PepT1 along the crypt-villus axis in intact tissue from WT and PepT1 KO was confirmed by immunofluorescence (Fig. 3e and Supplementary Fig. 2a–c).

### Expression of miRNAs in crypt and villi epithelial cells from WT and PepT1 KO mice

To investigate the miRNA expression profiles of intestinal epithelial cells (IEC) along the crypt-villus axis in WT and PepT1 KO mice, miRNA microarrays were analyzed in WT and PepT1 KO villi and crypts in the IEC. Large numbers of miRNA were differentially expressed, with an average of 239 microRNAs detected per sample.

Levels of specific miRNAs were compared pairwise among WT and PepT1 KO villi and crypts. The miRNAs with differential *P* value < 0.05 and of signal strengths >500 were selected. Of the 239 targets assayed, 36 miRNAs showed significant differences in at least one of the comparisons (Supplementary Fig. 3). The expression levels were further verified by qRT-PCR (Supplementary Fig. 4).

### miRNAs expressed differently along crypt-villus axis in WT mice

**Figure 4:**
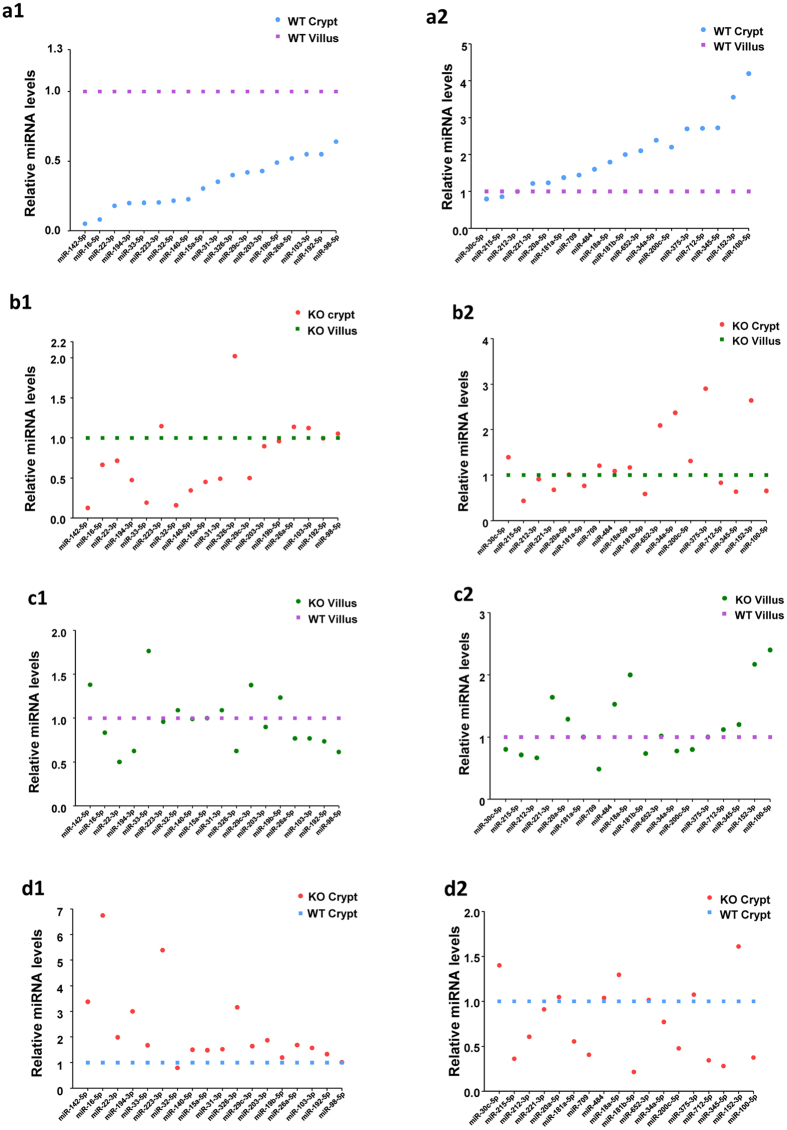
Expression levels of miRNAs in crypt and villus epithelial cells from BL-6 WT and PepT1 KO mice. A total of 36 miRNA transcripts were found to be differentially expressed in the villi and crypts of BL-6 WT and PepT1 KO mice; those having *P* < 0.05 and signal >500 are shown (n = 4). (**a1**,**a2**) In BL-6 WT crypts compared to BL-6 WT villi, 20 miRNAs were lower expressed (blue spots below purple spots), 1 displayed similar expression levels, and 15 were higher expressed (blue spots above purple spots). (**b1**,**b2**) In PepT1 KO crypts compared to PepT1 KO villi, 17 miRNAs were lower expressed (red spots below green spots), 12 displayed similar expression levels in the two tissues, and 7 were higher expressed (red spots above green spots). (**c1**,**c2**). In PepT1 KO villi compared to BL-6 WT villi, 15 miRNAs were down-regulated (green spots below purple spots), 10 displayed similar expression levels, and 11 were up-regulated (green spots above purple spots). (**d1**,**d2**) In PepT1 KO crypts compared to BL-6 WT crypts, 11 miRNAs were down-regulated (red spots below blue spots), 11 displayed similar expression levels, and 14 were up-regulated (red spots above blue spots).

Of the 36 differentially expressed miRNAs, 20 were down-regulated in WT crypts compared with WT villi (Fig. 4a1, with all 18 blue spots below the purple spots, and Fig. 4a2, with the first two blue spots below the purple spots), 1 miRNA (miR-212-3p) showed the same level of expression in WT villi and crypts (Fig. 4a2; the third blue spot, overlapping the purple spot), and 15 miRNAs were up-regulated in WT crypts relative to villi (Fig. 4a2, with blue spots 4 to 18 above the purple spots). Of the 20 miRNAs downregulated in crypts, 8 showed a >4.0 fold difference (miR-142-5p, miR-16-5p, miR-22-3p, miR-194-3p, miR-33-5p, miR-223-3p, miR-32-5p, miR-140-5p; Fig. 4a1, blue spots), whereas, of the 15 miRNAs upregulated in crypts, 2 showed a >3.0 fold difference (miR-192-5p, miR-98-5p) (Fig. 4a2, blue spots). Together these results demonstrate that miRNAs are differentially expressed along the crypt-villus axis in WT mice.

### PepT1 expression affects the normal miRNA distribution along crypt-villus axis

The expression levels of the 36 differentially expressed miRNAs were also compared in PepT1 KO crypts and villi (Fig. 4b). In contrast to WT mice, 17 miRNAs were down-regulated in PepT1 KO crypts compared with PepT1 KO villi (Fig. 4b1,b2, with red spots below the green spots), 12 miRNAs demonstrated same expression level (Fig. 4b1,b2, with red spots overlapping green spots), and 7 miRNAs were up-regulated in PepT1 KO crypts compared with PepT1 KO villi (Fig. 4b1,b2 red spots above the green spots). Specifically, 5 miRNAs (miR-223-3p, miR-326-3p, miR-26a-5p, miR-103-3p, miR-98-5p; Fig. 4a1 Blue spots vs Fig. 4b1: Red spots) showed expression profiles along the crypt-villus axis in PepT1 KO opposite to those in WT mice. 6 miRNAs (miR-221-3p, miR-181-5p, miR-181b-5p, miR-712-5p, miR-345-5p, miR-100-5p; Fig. 4a2,b2) showed a lower expression level in KO crypts than KO villi, but showed higher expression level in WT crypts than WT villi (Fig. 4b2: Red spots vs Fig. 4a2: Blue spots). Together, these results demonstrate that knockout of PepT1 markedly alters normal miRNA distribution along the crypt-villus axis compared with WT mice (Fig. 4b1,b2 vs Fig. 4a1,a2).

### PepT1 expression altered miRNA profiles in both villi and crypts

Comparisons of miRNA expression in PepT1 KO and WT villi showed that levels of 15 miRNAs were lower in villi from PepT1 KO mice than that from WT mice (Fig. 4c1,c2; green spots below the purple spots), whereas levels of 11 miRNAs were higher in villi from PepT1 KO than from WT mice (Fig. 4c1,c2; green spots above the purple spots). In addition, the expression levels of miRNAs also differed in crypts from PepT1 KO and WT mice. Specifically, 11 miRNAs were less expressed (Fig. 4d1,d2; red spots below blue spots) and 14 miRNAs were more expressed (Fig. 4d1,d2; red spots above blue spots) in crypt cells from PepT1 KO mice than in crypt cells from WT mice (Fig. 4d).

### Overall distribution of miRNAs in crypt and villus cells from PepT1 KO and WT mice

**Figure 5:**
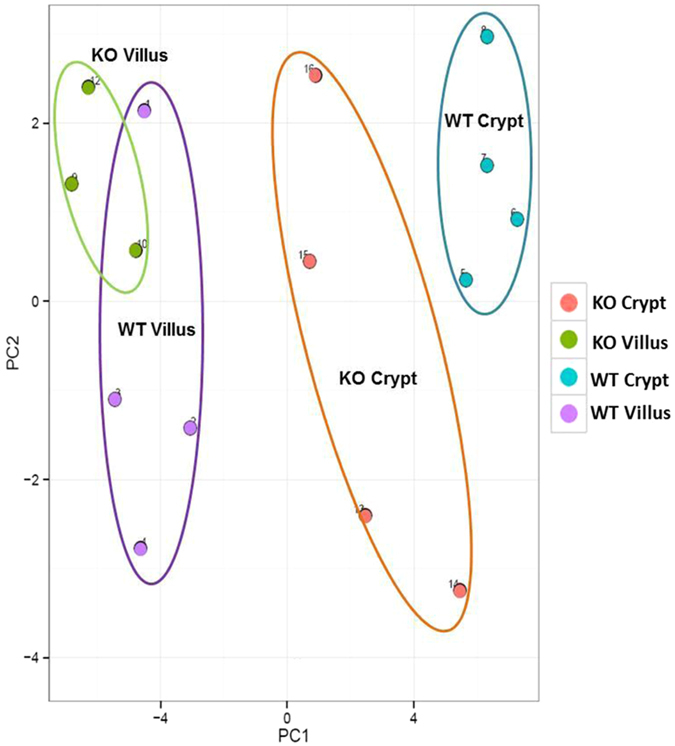
Overall distribution of miRNAs in crypt and villus cells from PepT1 KO and BL-6 WT mice. Overall miRNA populations are shown as a Principal Component Analysis (PCA) plot including the top 50 miRNAs that showed the largest variations across all samples.

The overall distribution of miRNAs was further confirmed by principal component analysis (PCA), which demonstrates overviews of sample clusters based on the 50 miRNAs with the largest variations across all samples (Fig. 5). The largest component in the variation is plotted along the X-axis and the second largest is plotted on the Y-axis. PCA analysis revealed that 15 samples can be roughly divided into 4 groups. miRNAs from PepT1 KO and WT villi were distinct from miRNAs from KO and WT crypts (Fig. 5). Thus, in both PepT1 KO mice and their WT controls, there were significant differences in miRNA expression profiles between villi and crypts.

### Proteins are differently expressed along crypt-villus axis in WT mice

In order to investigate whether miRNA alteration were accompanied by changes of protein expression, we further analyzed protein accumulation along the crypt-villus axis. Differential protein expression in crypt and villus cells from WT mice were analyzed by two-dimensional difference gel electrophoresis (2D-DIGE), as described in Methods section. Analysis of gel images showed 43 spots with >2.0-fold differences in spot intensity between WT villi and crypts (Supplementary Fig. 5a). Of these 43 spots, 18 were up-regulated, and 25 were down-regulated in crypts relative to villi. 18 candidate spots were picked from the total 43 spots based on their reliability, location on the gel, intensity, and fold change (>2.24) for mass spectrometry.

**Figure 6:**
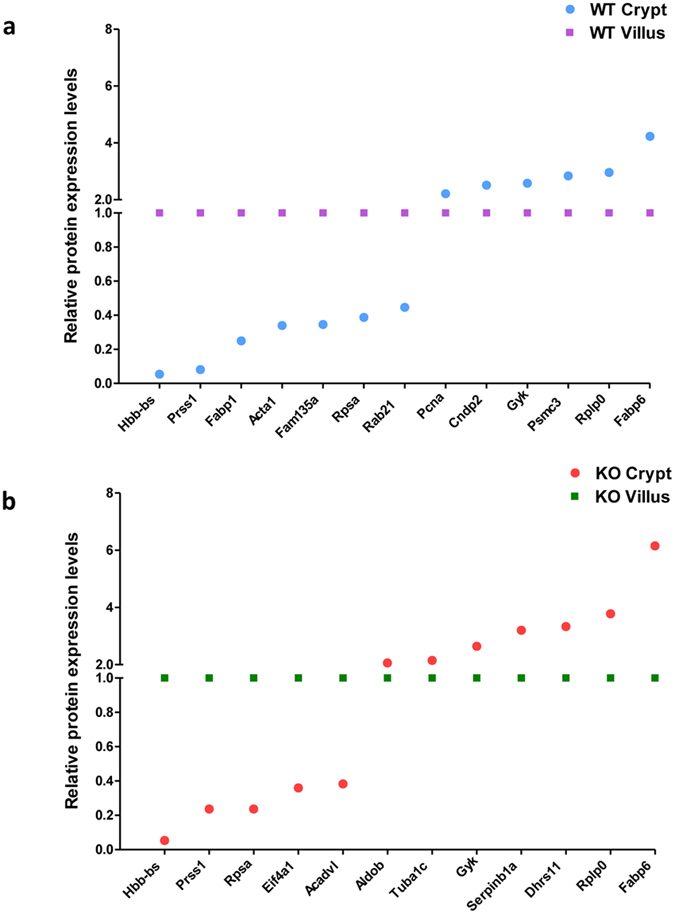
Differential expression of proteins along the crypt-villus axis. (**a**) In BL-6 WT crypts compared to BL-6 WT villi, 7 proteins were lower expressed (blue spots below purple spots) and 6 proteins were higher expressed (blue spots above purple spots). (**b**) In PepT1 KO crypts compared to PepT1 KO villi, 5 proteins were lower expressed (red spots below green spots) and 7 proteins were higher expressed (red spots above green spots).

To identify these selected spots, LC-MS was used, and 13 different proteins were identified. Of these, 7 proteins, Protein Hbb-bs (Hbb-bs), trypsin 1 (Prss1), fatty acid-binding protein (Fabp1), skeletal muscle alpha-actin mRNA (Acta1), family with sequence similarity 135 (Fam135a), laminin receptor (Rpsa), and Ras-related protein Rab-21 (Rab-21), exhibited significantly lower expression levels in WT crypts than in WT villi (Fig. 6a, blue round spots below the purple square spots). 6 other proteins, proliferating cell nuclear antigen (Pcna), cytosolic non-specific di-peptidase (Cndp2), glycerol kinase (Gyk), 26S protease regulatory subunit 6A (Psmc3), 60S acidic ribosomal protein P0 (Rplp0), and gastrotropin (Fabp6), showed higher expression levels in WT crypt than in WT villus cells (Fig. 6a, blue round spots above purple square spots). Spot number, GI accession number, gene symbol, protein name, protein molecular weight, isoelectric point (PI), average fold change, and overall trend of these spots are shown in Table 1. Thus, using 2D-DIGE/mass spectrometry on isolated crypt and villus samples identified proteins with different expression levels along the crypt-villus axis in WT mice.

### PepT1 expression disturbs normal differential protein expression along crypt-villus axis

2D-DIGE was also used to assess differential protein expression in the crypt and villus cells from PepT1 KO mice. Gel image analysis identified 72 spots with a >2.0-fold difference in spot intensity between PepT1 KO villi and crypts (Supplementary Fig. 5b). Of these 72 spots, 28 were up-regulated and 44 were down-regulated in crypts relative to villi of PepT1 KO mice. 17 candidate spots were picked based on their reliability, location on the gel, intensity, and fold change (>2.15) for mass spectrometry.

LC-MS of the 17 spots identified 12 different proteins. Of these, 5 proteins, Hbb-bs, Prss1, Rpsa, eukaryotic translation initiation factor 4Aa1 (Eif4a1), and very long chain specific acyl-CoA dehydrogenase, mitochondrial (Acadvl), demonstrated lower expression levels in PepT1 KO crypts than in PepT1 KO villi (Fig. 6b; red round spots below green square spots). 7 other proteins, fructose-bisphosphate aldolase (Aldob), tubulin alpha-1C chain (Tuba1c), Gyk, Fabp6, leukocyte elastase inhibitor A (Serpinb1a), dehydrogenase (Dhrs11), and Rplp0, showed higher expression levels in PepT1 KO crypts than in PepT1 KO villi (Fig. 6b; red round spots above green square spots). Spot number, GI accession number, gene symbol, protein name, protein molecular weight, PI, average fold change, and overall trend of these spots are shown in Table 2.

Comparison of the differently expressed proteins along the crypt-villus axis in WT and PepT1 KO mice showed that PepT1 knockout had no effect on the expression of Hbb-bs, Prss1, Rpsa, Gyk, Rplp0, and Fabp6. Although Fabp1, Acta1, Fam135a, Rab21, Pcna, Cndp2, and Psmc3 were differently expressed along the crypt-villus axis in WT mice (Fig. 6a and Table 1), they showed similar expression levels along this axis in PepT1 KO mice (these proteins are not shown in Fig. 6b and Table 2 because of their similar expression levels along the crypt-villus axis in PepT1 KO mice). Several other proteins, Eif4a1, Acadvl, Aldob, Tuba1c, Serpinb1a, and Dhrs11, had similar expression levels along the crypt-villus axis in WT mice (these proteins are not shown in Fig. 6a and Table 1 because of their similar expression levels different along the crypt-villus in WT mice), but were differentially expressed along this axis in PepT1 KO mice (Fig. 6b and Table 2). Together, these results demonstrate that PepT1 expression dysregulates the normal differential expression of proteins along the crypt-villus axis.

### PepT1 expression altered normal protein profile in villus cells

**Figure 7:**
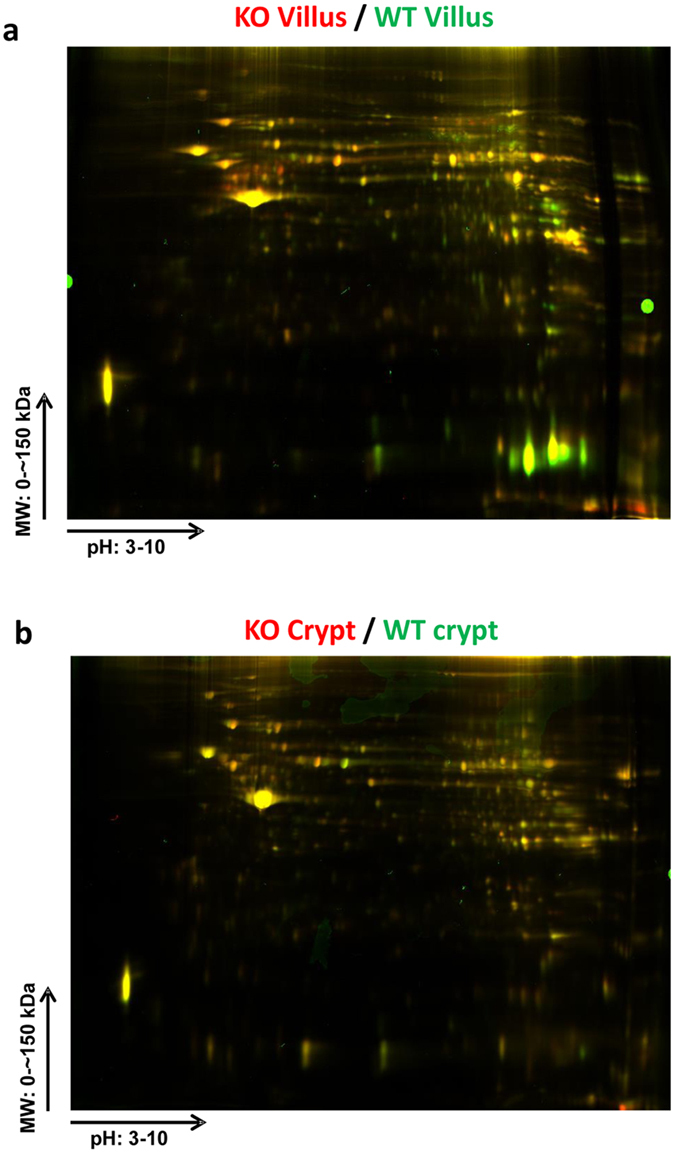
2D-DIGE-based assessment of differential protein expression. (**a**) Superimposed 2D-DIGE overlay image of protein accumulation in the villi of PepT1 KO (Cy5, red fluorescence) and BL-6 WT (Cy3, green fluorescence) mice. (**b**) Superimposed 2D-DIGE overlay image of protein accumulation in the crypts of PepT1 KO (Cy5, red fluorescence) and BL-6 WT (Cy3, green fluorescence) mice.

To investigate the effects of PepT1 knockout on protein expression in villus cells, protein expression was compared between PepT1 KO villi and WT villi. Representative 2D-DIGE gel images are shown in Fig. 7a. Relative to WT, 14 proteins were up-regulated and 10 were down-regulated in PepT1 KO villi (Supplementary Fig. 6a). 13 candidate spots were picked based on their reliability, location on the gel, intensity, and fold change (>2.09) for mass spectrometry.

**Figure 8:**
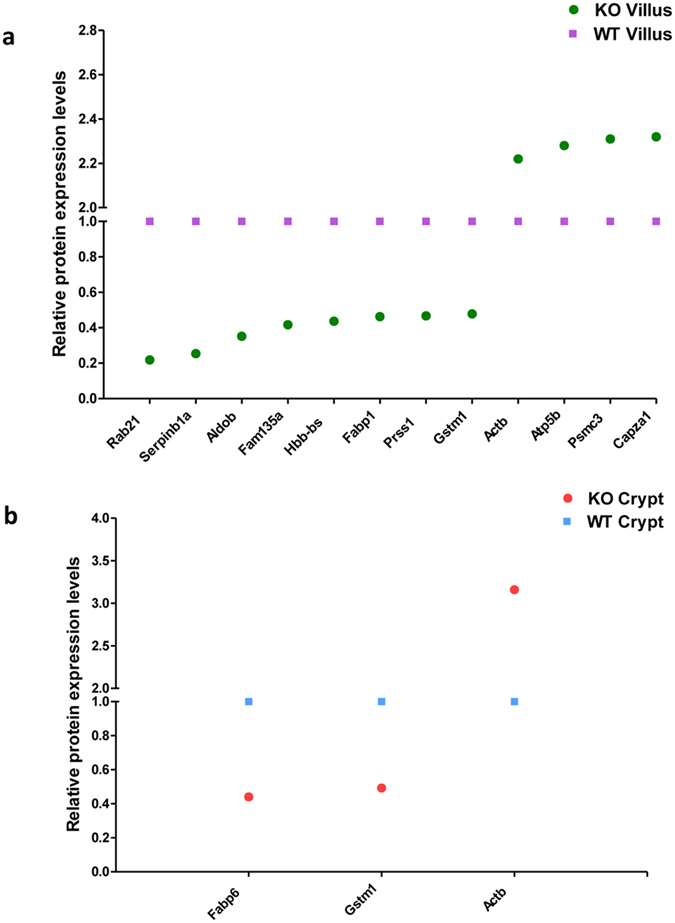
The protein expression gradient differs along the crypt-villus axis of PepT1 KO and BL-6 WT mice. (**a**) In PepT1 KO villi compared to BL-6 WT villi, 8 proteins were down-regulated (green spots below purple spots) and 4 proteins were up-regulated (green spots above purple spots). (**b**) In PepT1 KO crypts compared to BL-6 WT crypts, 2 proteins were down-regulated (red spots below blue spots) and 1 protein was up-regulated (red spots above blue spots).

**Figure 9:**
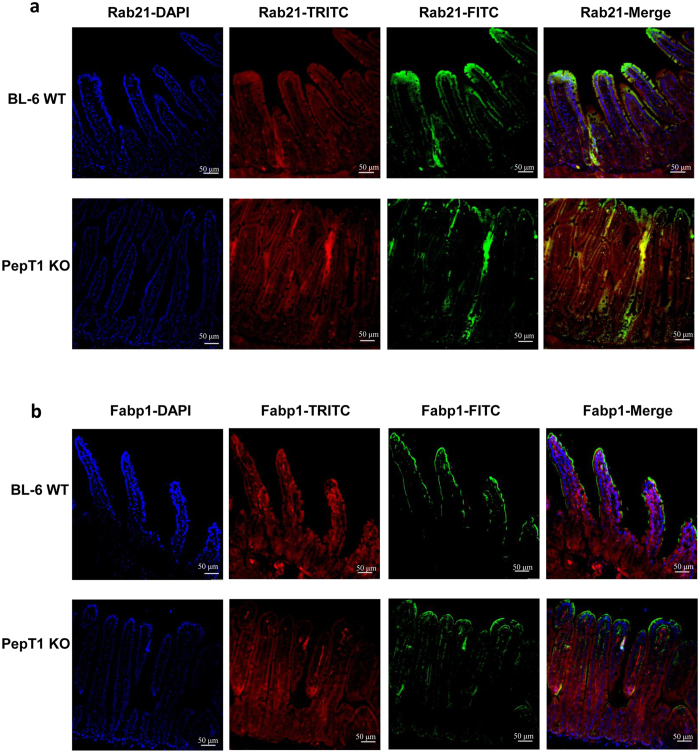
PepT1 KO alters the protein expression levels of Rab21 and Fabp1. (**a**) The expression of Rab21 was assessed by immunofluorescence. Rab21 was immunostained using anti-Rab21 (FITC, green), F-actin was stained using phalloidin (TRITC, red), and cell nuclei were stained using DAPI (blue). (**b**) The expression of Fabp1 was assessed by immunofluorescence. Fabp1 was immunostained using anti-Fabp1 (FITC, green), F-actin was stained using phalloidin (TRITC, red), and cell nuclei were stained using DAPI (blue). Separate pictures were taken at 20× for each filter, and the images were merged. Scale bar, 50 μm.

12 proteins were identified from the 13 selected spots using LC-MS. The expression levels of Rab-21, Serpinb1a, Aldob, Fam135a, Fabp1, Hbb-bs, Prss1, and glutathione S-transferase Mu1 (Gstm1) were lower in PepT1 KO than in WT villi (Fig. 8a: green spots below the purple squares, Fig. 9: Immunofluorescence staining showing lower expression of Rab-21 and Fabp1 in PepT1 KO than in WT villi); whereas the expression levels of Actb, ATP synthase subunit beta, mitochondrial (Atp5b), Psmc3, and capping protein muscle Z-line, alpha 1 (Capza1) were higher in PepT1 KO villi than in WT villi (Fig. 8a: green spots above the purple squares). Spot number, GI accession number, gene symbol, protein name, protein molecular weight, PI, average fold change, and overall trend are shown in Table 3. Together, these results demonstrate that PepT1 knockout modified normal protein expression in villus cells.

### PepT1 expression altered normal protein profile in crypt cells

To investigate the effects of PepT1 KO on protein expression in crypts, protein expression levels were compared between PepT1 KO and WT crypts. Representative 2D-DIGE gel images are shown in Fig. 7b. 2 proteins were up-regulated and 3 were down-regulated with volume difference in PepT1 KO crypts (Supplementary Fig. 6b). 3 candidate spots were picked based on their reliability, location on the gel, intensity, and fold change (>2.03) for mass spectrometry.

LC-MS identified 3 proteins. The levels of Fabp6 and Gstm1 were lower (Fig. 8b: red round spots below blue square spots), while the levels of Actb were higher (Fig. 8b: red round spots above blue square spots), in PepT1 KO than in WT crypts. Spot number, GI accession number, gene symbol, protein name, protein molecular weight, PI, average fold change, and overall trend are shown in Table 4. Together these results demonstrate that PepT1 knockout modified normal protein expression in crypt cells.

**Figure 10:**
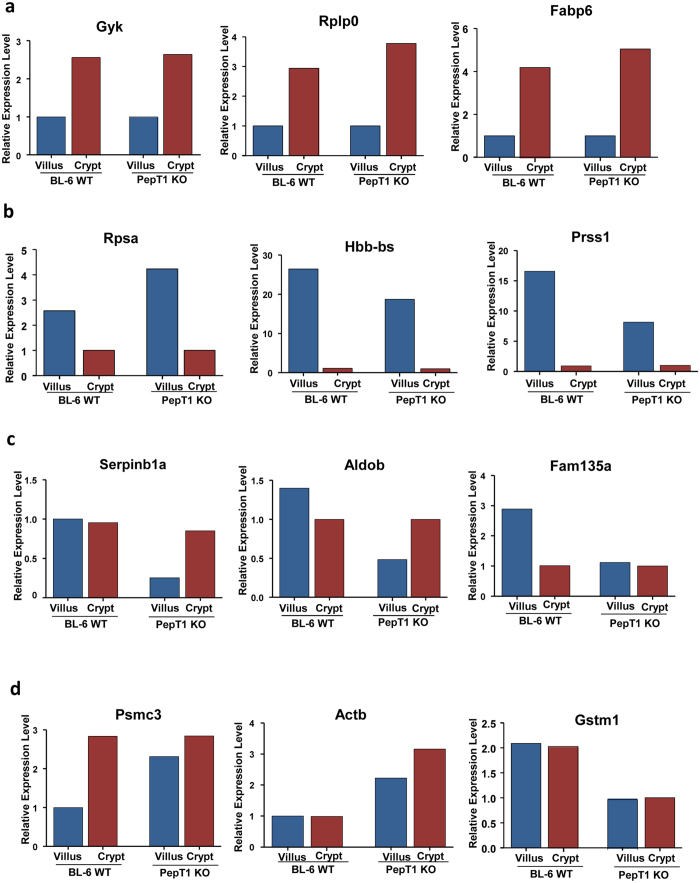
PepT1 KO alters the profiles of specific proteins along the crypt-villus axis. (**a**,**b**) The expression gradients of Gyk, Rplp0, Fabp6 (shown in *A*), Rpsa, Hbb-bs, and Prss1 (shown in B) along the crypt-villus axis are similar in PepT1 KO and BL-6 WT mice. (**c**,**d**) The protein expression gradients of Serpinb1a, Aldob, Fam135a, Psm3, Actb and GStm1 differ between PepT1 KO and BL-6 WT mice.

Collectively, these results demonstrate that 3 proteins, Gyk, Rplp0, and Fabp6, show the same expression gradient along the crypt-villus axis in both PepT1 KO and WT mice, with much higher expression levels in crypts than in villi in both WT and PepT1 KO mice (Fig. 10a). 3 other proteins, Rpsa, Hbb-bs, and Prss1, were less expressed in crypts than in villi in both WT and KO mice (Fig. 10b). However, the protein expression gradients of Serpinb1a, Aldob, Fam135a, Psm3, Actb and GStm1 differed between PepT1 KO and WT mice (Fig. 10c,d). Together, these results demonstrate that protein express differently along the crypt-villus axis, and knockout of PepT1 alters some of the protein expression gradient along this axis.

### PepT1 alters miRNA expression and their target protein expression

The potential target genes of the 36 selected miRNAs were determined by 3 different algorithms, as described in the Materials and Methods section. The proteins identified on 2D-DIGE were examined in the target list for each miRNA. As mentioned in the Introduction, miRNAs and their target proteins showed reverse associations. Thus, we filtered the proteins that could be a target of a certain miRNA and showed reverse expression compared with that of miRNA.

**Figure 11:**
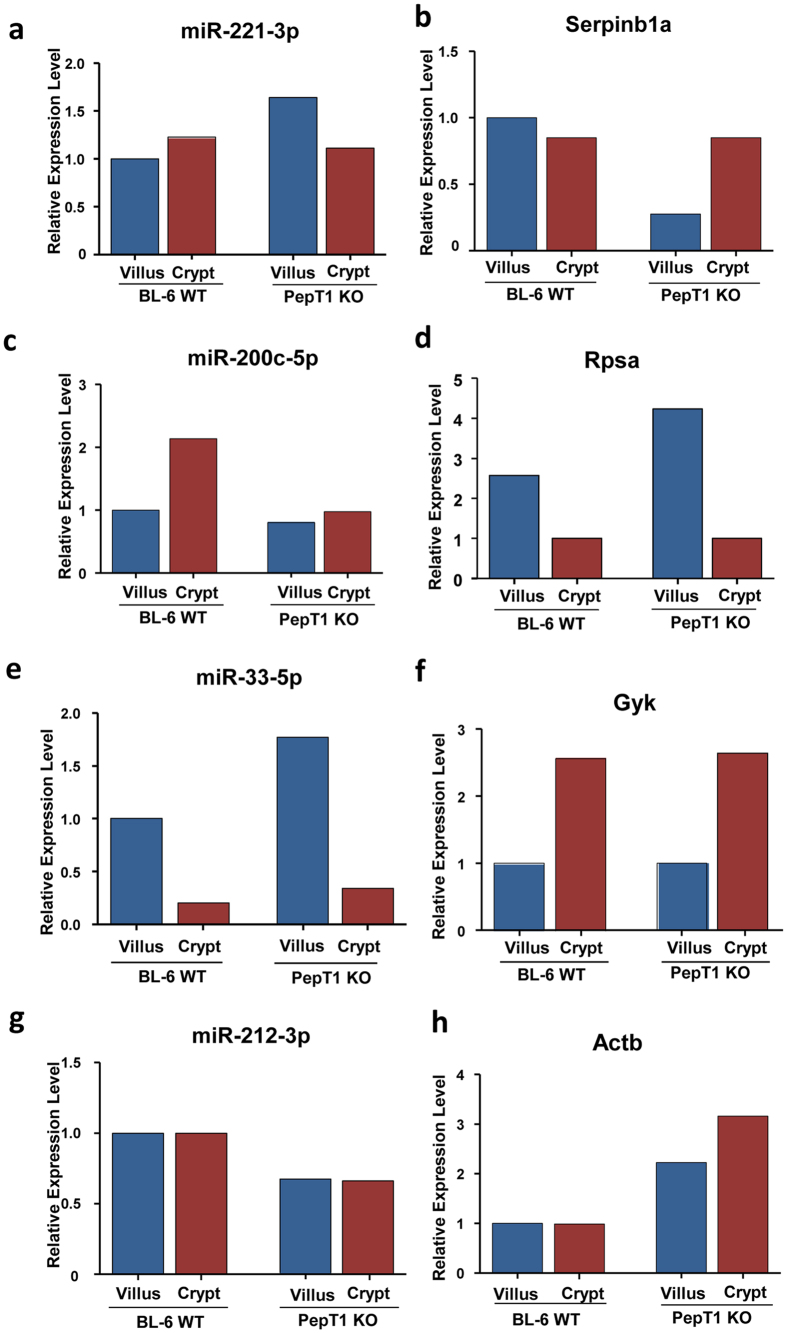
PepT1 KO modulates the expression levels of certain miRNAs, thereby altering the expression levels of their target proteins. (**a**,**b**) In BL-6 WT mice, miRNA-221-3p was higher in crypts than in villi. In PepT1 KO mice, in contrast, this miRNA was higher in villi than in crypts. This same pattern was reflected in the expression of its target protein, Serpinb1a. (**c**,**d**) In BL-6 WT mice, miRNA-200c-5p was higher in crypts than in villi. This pattern was reflected in the expression of its target, Rpsa. (**e**,**f**) Corresponding gradient changes along the crypt-villus axis were seen in miRNA-33-5p and its target, Gyk in both BL-6 WT mice and PepT1 KO mice. (**g**,**h**) In both villi and crypts, miR-212-3p was higher in BL-6WT mice than in PepT1 KO mice, and this difference was reflected in the expression of its target, Actb.

4 pairs of miRNA and proteins were found to be related (Table 5). The expression of miRNA-221-3p was higher in crypts than in villi of WT mice, whereas it was higher in villi than in crypts of PepT1 KO mice (Fig. 11a). This change in expression gradient was also reflected in the level of its target protein Serpinb1a (Fig. 11b). Similarly, the expression of miR-200c-5p was higher in WT crypts than in WT villi, but the levels were similar in crypts and villi of PepT1 KO mice (Fig. 11c). These findings were reflected by the expression of its target protein Rpsa (Fig. 11d). Both miRNA-33-5p and its target protein Gyk showed the same gradient changes along the crypt-villus axis in WT and PepT1 KO mice (Fig. 11e,f). Both miR-212-3p and its target Actb showed no expression gradient along the crypt-villus axis in WT and PepT1 KO mice; however, the levels of expression of miR-212-3p were equivalently high in WT crypts and villi and equivalently low in PepT1 KO crypts and villi (Fig. 11g,h). The expression of its target protein Actb was in agreement with miR-212-3p expression (Fig. 11g,h). Together, these results suggest that knockout of PepT1 protein affects the expression of certain miRNAs, modifying the expression of their target proteins.

### PepT1 expression affects apoptosis and proliferation of IECs

**Figure 12:**
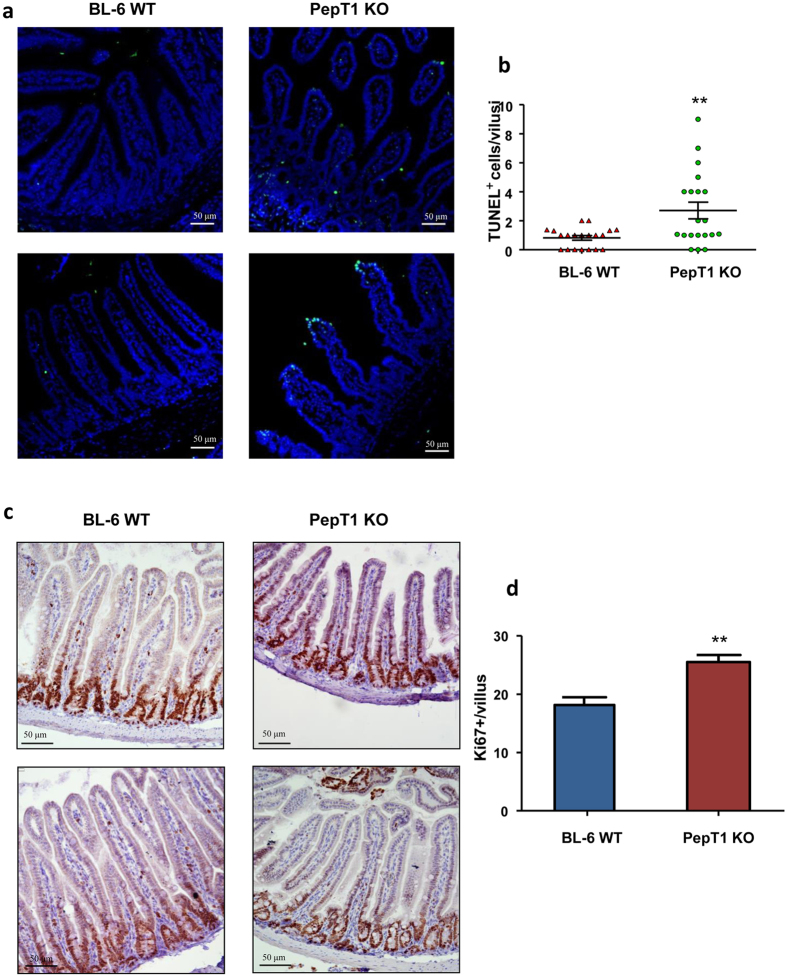
PepT1 KO increases small intestinal epithelial cell proliferation and apoptosis. (**a**) Apoptotic small intestinal epithelial cells were quantified using a TUNEL assay (FITC, green) and nuclei were stained with DAPI (blue). (**b**) Cells positive for both TUNEL and DAPI staining were counted and averaged per villus. (**c**) The levels of epithelial cell proliferation in small intestine sections from BL-6 WT and PepT1 KO mice were assessed by immunohistochemistry using the proliferation marker, Ki67. (**d**) Ki67^+^ cells were counted and averaged per villus. Scale bars, 50 μm; **P < 0.005.

We have shown that PepT1 expression altered morphology of crypt-villus axis and molecular distribution of miRNA and protein. Further, we wanted to know if these changes are accompanied to modification of normal tissue homeostasis. Normal tissue homeostasis is determined by the balance between apoptosis and cell proliferation. A key feature of intestinal homeostasis is the ability to maintain epithelial integrity and trigger cell renewal along the crypt-villus axis. Here, we used TUNEL and Ki67 staining of small intestinal sections to assess IEC apoptosis and proliferation. As shown in Fig. 12a, the apoptosis of IECs was higher in the villi of PepT1 KO mice than in those of WT mice. IEC proliferation was also increased in PepT1 KO mice compared to WT mice. Moreover, in contrast to WT mice, IEC proliferation was not largely restricted to crypt cells (Fig. 12b). These results indicate that PepT1 expression modulates the balance of apoptosis and cell proliferation in the small intestine, and is thus likely to affect the overall function of the small intestine.

## Discussion

We herein show that ablation of PepT1 expression does not significantly affect villus length in PepT1 KO mice. It does, however, affect the apoptosis and proliferation of IECs, suggesting that PepT1 contributes to maintaining intestinal homeostasis. The body weights of PepT1 KO mice were significantly lower than those of WT animals. This may reflect the decreased microvillus size observed in PepT1 KO mice, which would be likely to reduce their overall nutrient absorption and weight gain. These observations are in agreement with previous reports that mice lacking PepT1 exhibited reduced energy absorption^34^. We found that PepT1 expression exhibited a marked crypt-villus gradient. This is consistent with previous studies showing that PepT1 is abundant at the tip of the villus and decreases toward its base^35^. These observations show that PepT1 is specific to the plasma membranes of the differentiated absorptive epithelial cells that form microvilli. Thus, a lack of PepT1 expression may limit microvilli development and decrease the overall capacity for intestinal absorption^36^. These observations collectively suggest that PepT1 expression may affect major intestinal functions.

Protein expression along the crypt-villus axis is subject to a very dynamic regulatory process. The stem cells found at the base of the crypt continually divide and are the source of all epithelial cells^37^,^38^. Cell proliferation, lineage-specific differentiation, migration, and apoptosis and/or cell shedding are all tightly interrelated along the crypt-villus axis^39^. A number of factors have been shown to regulate cell fate and differentiation in the intestine^40^,^41^, and the overall gene expression pattern has been shown to differ between crypts and villi^42^.

miRNAs play critical roles in important biological processes, including development, differentiation, proliferation, and apoptosis^43^,^44^,^45^. Our group previously showed that specific miRNA expression profiles are associated with the different differentiation statuses of IECs, and that miRNAs could contribute to determining the unique physiological characteristics of human IECs^46^. Thus, we speculated that miRNAs could be involved in highly regulated process of protein expression/repression along the crypt-villus axis.

It is well known that the combined activities of various transcription factors modulate the expression of transcriptional targets in IEC along the crypt-villus axis^47^. Transcription factors are differentially expressed along the crypt and villus, presumably forming a basis for the differential protein expression between these cell types. It has been reported that miRNA expression is cell-type-specific^48^. However, the miRNA expression levels in the various intestinal epithelial cell subtypes have not previously been studied along the crypt-villus axis.

Here, we describe cell fractionation experiments using mouse small intestinal epithelial cells, and report that miRNAs are differently expressed along the crypt-villus axis, where they are likely to play important roles in the expression/repression of various proteins, including transcription factors. We suggest that proper specification of the intestine during development relies on not only the action of transcription factors, but also the effects of these miRNAs. Moreover, we propose that tight control of the miRNA expression gradients along the crypt-villus axis is crucial for the maintenance of normal intestinal epithelial integrity^49^.

To evaluate the potential interdependence of protein and miRNA expression levels along the crypt-villus axis, we used the PepT1 KO mice model. In our previous study, we demonstrated that overexpression of PepT1 in a specific tissue (colon) altered the overall miRNA expression in that tissue^27^. In the present study, we observed that KO of PepT1, which is mostly expressed in villi, affected the expression levels and expression gradients of miRNAs in the small intestine. Interestingly, we observed that the lacking of PepT1 expression affect the normal miRNAs expression along the crypt-villus axis of WT mice. When comparing the same miRNAs, we observed a change of the miRNAs expression in crypt cell in mice lacking PepT1 expression in crypt cells. The later observation implies that PepT1 knockout could therefore conceivably dysregulate protein expression in crypt cells *via* the observed alterations in miRNA expression or through other miRNAs-independent pathways. Thus, PepT1 expression could impact the regulatory networks of crypt cells at least in part by modulating the steady-state levels of miRNAs in these cells. Consistent with this proposal, a recent study exhibited that viruses such as HIV disrupt normal host miRNA expression specifically in the proliferative crypt region. Notably, proper miRNA expression in this region controls the expression levels of genes involved in cell death and epithelial maturation, and thereby affects overall intestinal homeostasis^50^. The PepT1 KO mice also exhibited disrupted miRNA expression profiles in their villus cells. We do not yet know whether this is a downstream effect of altered miRNA biogenesis in the crypt cells or a direct effect on villus cells.

We further observed that the protein expression gradients along the crypt-villus was altered in PepT1 KO mice compared to WT, suggesting that the homeostasis of the small intestine was affected in PepT1 KO mice. The altered proteins included Ras-related protein (Rab21), which belongs to a subfamily of Ras-superfamily small GTP-binding proteins. Rab21 plays an important role in regulating vesicular transport at the apical sides of polarized intestinal epithelial cells^51^, and acts as a signal transduction molecule in the development of the brush borders (microvilli)^52^. In PepT1 KO mice, Rab21 expression was ~4.58-fold lower than that in WT mice. This could explain why the microvilli in PepT1 KO mice are less developed than those of WT mice, which would affect intestinal absorption in these mice (Fig. 9a). Another altered protein was fatty acid-binding protein (Fabp1), which shows high-level expression in villus cells but low-level expression in crypt cells of normal mice, and is used as a marker of cell differentiation^53^. We observed that Fabp1 expression was reduced in the villus cells of PepT1 KO mice, suggesting that such mice may exhibit altered cell differentiation along the crypt-villus axis (Fig. 9b).

We herein report that PepT1 KO affects the expression levels and gradients of both miRNAs and proteins along the crypt-villus axis, and that there are correlations between the changes in miRNA and protein levels. For example, miR-221-3p and its protein target, serpinb1a, are expressed equally in the crypt and villus cells of WT mice but exhibit gradients between the crypts and villi of PepT1 KO mice. These results support our hypothesis that the absence of PepT1 expression dysregulates normal miRNA expression and alters the expression levels of their target proteins along the crypt-villus axis. The latter observation implies that PepT1 KO could conceivably dysregulate protein expression in the crypt-villus axis via the observed alterations in miRNA expression. However, we cannot rule out that the observed dysregulated protein expression along the crypt-villus axis in PepT1 KO could also be miRNAs-independent. To determine whether this correlation between miRNA expression and target protein expression is direct, a transfect of certain miRNAs into isolated crypts or enteroids from WT and KO mice could be helpful and need to be further studied.

We found that PepT1 and the differential expression of its target miRNAs along the crypt-villus axis could contribute to cell fate decisions along the crypt-villus axis. Previous studies have shown that transcription factors are required for proper specification of cells along the crypt-villus axis^54^. Based on our present results, we demonstrate that miRNAs could post-transcriptionally modulate or fine-tune the dynamics of the cellular processes that occur along this axis. The disruption of normal miRNA expression along the axis, such as that seen in some pathologies like miRNA-135b overexpression in colon cancer^55^, may affect intestinal homeostasis and/or functions. Our results also suggest that PepT1 helps maintain small intestinal homeostasis and function by regulating miRNA/protein expression along the crypt-villus axis. These alterations could occur *via* the actions of PepT1 on transcription factors and/or miRNAs, or indirect through other pathways including other cellular components or immune cells, offering multiple combinations through which different cellular functions may be regulated.

Although PepT1 is found to be mostly expressed in the small intestine, low levels of PepT1 expression is also reported in the kidney, pancreas and bile duct^18^,^19^,^20^. In the kidney, PepT1 is detected in the cortex. Since another oligopeptide transporter PepT2 has greater abundance over PepT1 in kidney, we speculate that peptides are predominantly reabsorbed in kidney by PepT2^18^. However, we cannot rule out the possibility that ablation of PePT1 in kidney or other organs will also play important roles in mediating their homeostasis.

## Methods

### Mouse model

PepT1 KO mice obtained from Deltagen (San Mateo, CA) were backcrossed with WT (C57BL/6) animals to obtain the same genetic background in both controls (WT and PepT1 KO). Genomic DNA from tail snips was extracted using the RED Extract-Amp Tissue PCR Kit (Sigma, St Louis, MO) according to the manufacturer’s protocol. The primers used for identifying PepT1 KO mice were 5′-GGGCCAGCTCATTCCTCCCACTCAT-3′, 5′-AGTGTGGGCTGGTGTGAGACACGTGT-3′ (forwards) and 5′-CAGGGGGAGAGAGAAACAGAGTTAG-3′ (reverse). Specific PCR products for each target gene were obtained under the following conditions: 94 °C for 3 min, 94 °C for 15 s, 55 °C for 30 s, 72 °C for 1 min, and 72 °C for 10 min for a total of 40 cycles. All mice were housed in groups of 5 per cage at Georgia State University under controlled conditions of 12:12 h dark/light, 5% humidity and 25 °C. Animal experiments were approved by the Institutional Animal Care and Use Committee of Georgia State University (Atlanta, GA), and performed in accordance with the guide for the Care and Use of Laboratory Animals by U.S. Public Health Service. All procedures were approved and registered in the protocol IACUC ID: A14007.

### Isolation of epithelium from crypts and villi of small intestine

Epithelium was isolated from crypts and villi of the small intestines of 8 week-old WT and PepT1 KO female mice using the low-temperature method^30^. Small intestines were cleaned and sliced into 2–3 mm sections. Pieces were washed in HBSS with 0.5 mM DTT for 5 min at 4 °C with constant stirring at 200 rpm (Step 1). Detached tissues were collected as sample 1. Tissue pellets were transferred to 115 ml of chelating buffer (27 mM trisodium citrate, 5 mM Na_2_HPO_4_, 96 mM NaCl, 8 mM KH_2_PO_4_, 1.5 mM KCl, 0.5 mM DTT, 55 mM D-sorbitol, 44 mM sucrose, pH 7.3), incubated at 4 °C for 20 min with constant stirring at 200 rpm, and re-collected as sample 2 (Step 2). The remaining pellets were re-suspended in 20 ml of fresh chelating buffer in a 50 ml centrifuge tube for washing. The tube was manually inverted 60 times and detached pieces were collected as sample 3 (Step 3). Next, 20 ml of fresh chelating buffer was added to the wash tube and manually washed for three times. Detached pieces were collected every time as sample 3 (Step 4). After manual washing, step 2 through step 4 was repeated three times to collect sample 4–9. Tissue pellets were further re-suspended in 20 ml chelating buffer in a 50 ml centrifuge tube and vigorously shaken by hand for 1 min. Detached pieces were collected as sample 10 (Step 5). Step 5 was then repeated to collect sample 11. Samples 1–11 collected from the separation procedures were re-suspended in 1 ml HBSS for further experimental use. The purity of villi and crypts was examined *via* microscopy and RT-PCR, and samples were stored at −80 °C until use. See Fig. 2.

### Sample collection and preparation

Approximately 2.0 cm pieces of small intestine or villi/crypt samples collected from isolated small intestine were homogenized in RIPA buffer (150 mM NaCl, 0.5% sodium deoxycholate, 50 mM Tris-HCl, pH 8.0, 0.1% SDS, 0.1% Nonidet P-40) with one tablet of protease inhibitor. Homogenates were centrifuged at 12,000 rpm for 10 min at 4 °C, and protein concentrations were measured using the DC protein assay kit (Bio-Rad, Hercules, CA). The protein solution was used either for Western Blot or 2-D analysis after 2-D clean-up. For 2-D clean-up, 230 mg of protein was precipitated, purified and cleaned using a specific kit (GE Healthcare Life Science, Piscataway, NJ), according to the manufacturer’s protocol. Precipitated pellets were re-suspended in 65 μl of rehydration buffer (2 M Thiourea, 7 M Urea, 4% Chaps, 25 mM Tris-HCl, pH 8.8).

### Sample labeling and two-dimensional gel electrophoresis

WT villi (or WT crypts) sample (30 μg) was labeled with 200 pmol N-hydroxysuccinimidyl-ester of cyanine dye, Cy3, and 30 mg KO villi (or KO crypts) sample was labeled with 200 pmol N-hydroxysuccinimidyl-ester of cyanine dye, Cy5 (GE Healthcare Life Science, Piscataway, NJ). After quenching with 10 mM lysine, the labeled proteins were mixed. Sample buffer (7 M urea, 4 M thiourea, 4% CHAPS, 2% IPG buffer, pH 4–11, NL) and rehydration solution (7 M urea, 4 M thiourea, 4% CHAPS, 1% DTT, 1% IPG) were added to a final volume of 350 μl for each gel. First-dimension isoelectric focusing (IEF) was performed using 24 cm IPG strips (pH 4–7, GE Healthcare Life Sciences, Piscataway, NJ) in EttanIPGphor (GE Healthcare Life Sciences, Piscataway, NJ). Strips containing samples were equilibrated, reduced, alkylated and stained *via* sequential incubation in 1.5% DTT equilibration buffer (50 mM Tris-HCl, 6 M urea, 30% glycerol, and 2% SDS) and 4.5% iodoacetamide equilibration buffer stained with bromophenol blue for 20 min each. Second-dimension electrophoresis was conducted on a 10% SDS polyacrylamide gel in the Ettan DALT II system separation unit (GE Healthcare Life Science, Piscataway, NJ).

Gel images were further acquired on a Typhoon Trio (GE Healthcare) at the appropriate wavelengths for Cy3 and Cy5 dyes, and analyzed using DeCyder image analysis software (V7.0, GE Healthcare). Next, gels were visualized with colloidal Coomassie staining (SimplyBlue, Invitrogen, Carlsbad, CA). Coomassie-stained gel was re-scanned with the Typhoon Trio Scanner, and the image matched and aligned with Cy3 and Cy5 fluorescence images. A list of proteins of interest showing >1.75-fold increase or decrease was collated, based on the analysis. 3D views of individual spots generated with DeCyder image analysis software (GE Healthcare) were also considered while selecting the list of proteins.

### Spot picking and mass spectrometric protein identification

The pick list was exported to Ettan Spot Picker (GE Healthcare), and protein spots excised and transferred to a microtiter plate using Ettan Spot Picker. Picked gel pieces were washed initially with dd H_2_O and subsequently with washing solution I (50% ethanol, 10% acetic acid) following by washing solution II (50% acetonitrile, 100 mM ammonium bicarbonate, pH 8.3). Cleaned gel pieces were finally dehydrated with 100% acetonitrile and dried under a speed-vac. Dried gels were either digested with trypsin or incubated at −80 °C until trypsin treatment for mass spectrometry peptide analysis. Gel pieces were incubated with an appropriate amount of trypsin (Modified Trypsin Gold, Promega, Madison, WI) in ProteaseMax Surfactant (Promega, Madison, WI) at 37 °C for 2–3 h. After incubation, digested peptides were extracted with 2.5% trifluoroacetic acid, further purified and concentrated using ZipTip, a micro-reverse phase column (Millipore, Bilerica, MA), according to the manufacturer’s protocol. Extracted peptides were subsequently analyzed with a 4800 MALDI-TOF/TOF tandem mass spectrometer (AB Sciex, Framingham, MA) in the MS/MS tandem mode. Protein identification was accomplished using Mascot search engine (Matrix Science Inc, Boston, MA) against Swiss-Prot or NCBI protein databases.

### RNA extraction and real-time reverse transcription-PCR

Total RNA was extracted from mouse small intestine with the RNeasy Mini kit (Qiagen, Valencia, CA), according to the manufacturer’s instructions, and yield and quality verified. cDNA was generated from total RNA using the maxima first-strand cDNA synthesis kit (Thermo Scientific, Glen-Burnie, MD). cDNA of miRNAs was generated from total RNA preparations using NCode miRNA first-Strand cDNA synthesis and qRT-PCR kits (Invitrogen, Carlsbad, CA). Levels of targets were quantified with real-time reverse transcription (RT-PCR) using Maxima SYBR Green/ROX qPCR Master Mix (Fermentas). Fold induction was calculated using the Ct method as follows: 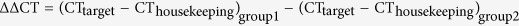 , and final data derived from 2^−ΔΔCT^.

### Microarray analysis of miRNA expression

Total RNA (50 ng) containing miRNA was extracted from small intestine of mice with the miRCURY RNA isolation kit-Tissue (Exiqon, Woburn, MA) according to the manufacturer’s protocol for purification of miRNA from animal tissue. RNA yield and quality were verified. All miRNA PCR reactions were performed by Exiqon Services, Vedvack, Denmark. RNA (10 ng) was reverse-transcribed in 50 μl reactions using the miRCURY LNA™ Universal RT microRNA PCR, Polyadenylation and cDNA synthesis kit (Exiqon). cDNA was diluted 100× and assayed in 10 μl reaction mixtures using the protocol for miRCURY LNA™ Universal RT microRNA PCR. Every microRNA was assayed once with qPCR on microRNA Ready-to-Use PCR, Mouse&Rat panel I+II using ExiLENT SYBR^®^ Green master mix. Negative controls, excluding the template from the reverse transcription reaction, were examined and profiled in a similar manner to samples. Amplification was performed in a LightCycler^®^ 480 Real-Time PCR System (Roche) in 384-well plates. Amplification curves were analyzed using Roche LC software, both for determination of Cq (with the second derivative method) and for melting curve analysis. Amplification efficiency was calculated using algorithms similar to those in LinReg software. Criteria for inclusion of an assay in the analysis included Cp less than 37 and 5 less than that of negative control. Data that did not satisfy these criteria were omitted from further analysis. All data were normalized to the average of samples detected in all assays (average – assay Cp), which was confirmed as the best normalizer using NormFinder.

### miRNA target prediction

To determine the potential target genes of detected miRNAs, miRSearch V3.0 algorithms (Exiqon, Woburn, MA) (https://www.exiqon.com/mirsearch), miRDB database (http://mirdb.org/cgi-bin/search.cgi), and TargetScanMouse (http://www.targetscan.org/mmu_61/) were used.

### Hematoxylin and eosin (H&E) staining

Mouse small intestines were fixed with 10%-buffered formalin for 24 h at room temperature and then embedded in paraffin. Tissues were sectioned at 6-μm and stained with H&E using standard protocols. Images were acquired using an Olympus microscope equipped with a DP-26 Digital camera (Olympus, Tokyo, Japan).

### Immunohistochemistry

Paraffin-embedded tissue sections were de-paraffinized in xylene and rehydrated in an ethanol gradient. For epitope retrieval, the sections were heated in 10 mM sodium citrate buffer (pH 6.0) for 10 min in a pressure cooker. The sections were then blocked with 5% goat serum in TBS and incubated for 1 hour at 37 °C with anti-PepT1 (1:200; Santa Cruz, Dallas, TX), anti-Muc2 (1:600; Santa Cruz, Dallas, TX), or anti-Rab21 (1:600; Abcam, Cambridge, MA). After being washed with PBS, the sections were incubated with Alexa-Fluor 568 phalloidin (1:5000; Invitrogen Carlsbad, CA) and a horseradish peroxidase-conjugated secondary antibody for 45 minutes at room temperature in the dark. Sections were mounted in mounting medium containing 4′, 6-diamidino-2-phenylindole (DAPI; Vector Laboratories, Burlingame, CA) and covered with coverslips. Images were acquired using an Olympus microscope equipped with a Hamamatsu Digital Camera ORCA-03G (Olympus, Tokyo, Japan).

### Terminal deoxynucleotidyl transferase deoxyuridine triphosphate nick-end labeling (TUNEL) staining

For quantification of apoptosis among epithelial cells of the small intestine, paraffin sections were de-paraffinized and stained for apoptotic nuclei using an *In Situ* Cell Death Detection Kit (Roche Diagnostics, Indianapolis, IN) according to the manufacturer’s instructions. Images were acquired using an Olympus microscope equipped with a Hamamatsu black and white ORCA-03G digital camera (Olympus, Tokyo, Japan). The numbers of TUNEL-positive cells that overlapped with DAPI nuclear staining were counted per villus.

### Ki67 staining

Mouse small intestinal tissues were formalin-fixed, paraffin-embedded, and sectioned. The sections were de-paraffinized in xylene and rehydrated in an ethanol gradient. For antigen retrieval, the sections were placed in 10 mM sodium citrate buffer (pH 6.0) and cooked with a pressure cooker for 10 minutes. The sections were blocked with 5% goat serum in TBS, and incubated with anti-Ki67 (1:100; Vector Laboratories, Burlingame, CA) for 1 hour at 37 °C. After being washed with TBS, the sections were treated with the appropriate biotinylated secondary antibodies for 1 hour at 37 °C, and color development was performed using a Vectastain ABC kit (Vector Laboratories, Burlingame, CA). The sections were then counterstained with hematoxylin, dehydrated, and coverslipped. Images were acquired using an Olympus microscope equipped with a DP-23 Digital camera (Olympus, Tokyo, Japan). The numbers of Ki-67-positive cells were counted per villus.

### Transmission electron microscopy (TEM)

Small intestine tissues were dissected into 1 to 2 mm cubes while immersed in 2.5% glutaraldehyde buffered with 0.1 M sodium cacodylate (pH 7.2). Samples were stored in the same fixative overnight at 4 °C. Samples were then washed with the same buffer and post-fixed in 1% buffered osmium tetroxide, dehydrated through a graded ethanol series to 100%, and embedded in Eponate 12 resin (Ted pella Inc., Redding, CA). Ultrathin sections were cut on a Leica UC6rt ultra microtome (Leica Microsystems, Buffalo Grove, IL) at 70–80 nm and counterstained with 4% aqueous uranyl acetate and 2% lead citrate. Sections were examined under a LEO906e transmission electron microscope (Carl Zeiss, Oberkochen, German) at a voltage of 80 kV.

### Western blot

Collected proteins (30 μg) were resolved on 12% polyacrylamide gels (Bio-Rad, Hercules, CA) and transferred to nitrocellulose membranes (Bio-Rad, Hercules, CA). Membranes were probed with anti-PepT1 primary antibody (dilution 1:1000; Santa Cruz, Dallas, TX). After three washes, membranes were incubated with the appropriate horseradish peroxidase-conjugated secondary antibodies (dilution 1:4000, GE Healthcare Biosciences, Pittsburgh, PA) and bands detected using the Enhanced Chemiluminescence Detection Kit (GE Healthcare Biosciences, Pittsburgh, PA).

### Statistical analysis

Values were expressed as means ± s.e.m. Statistical analysis for significance (*P* < 0.05) was determined using two-tailed Student’s *t*-test by GraphPad Prism 5 software. Differences were noted as significant with: *P < 0.05, **P < 0.005, ***P < 0.001.

## Additional Information

**How to cite this article**: Zhang, Y. *et al.* PepT1 Expression Helps Maintain Intestinal Homeostasis by Mediating the Differential Expression of miRNAs along the Crypt-Villus Axis. *Sci. Rep.* **6**, 27119; doi: 10.1038/srep27119 (2016).

## Supplementary Material

Supplementary Information

## Figures and Tables

**Table 1:** Identification of picked proteins differentially expressed between WT villus and WT crypt.

Spot No.^a^	Accession No.^b^	Gene^c^	Protein Name	Protein MW (kDa)	Protein PI	Average fold change^d^	Overall trend
1320	Q3TEN9	Gyk	Glycerol kinase	57.4	5.76	−2.58	Up
1400	NP_032974	Psmc3	26S protease regulatory subunit 6A	44.6	5.02	−2.84	Up
1800	B2CY77	Rpsa	Laminin receptor	32.8	4.87	2.58	Down
1952	P14869	Rplp0	60S acidic ribosomal protein P0	34.2	6.25	−2.96	Up
1961	Q61264	Acta1	Skeletal muscle alpha-actin mRNA	37.7	5.54	2.94	Down
2541	P35282	Rab21	Ras-related protein Rab-21	24.1	7.94	2.24	Down
2992	A0A087WQ08	Fam135a	Family with sequence similarity 135	30.5	7.01	2.89	Down
3086	Q9Z1R9	Prss1	protease, serine 1 (trypsin 1)	26.1	4.94	12.35	Down
3247	Q9D1A2	Cndp2	Cytosolic non-specific dipeptidase	52.7	5.66	−2.51	Up
3254	E9Q223	Hbb-bs	Protein Hbb-bs	11.1	6.37	18.52	Down
3261	P51162	Fabp6	Gastrotropin	14.5	6.15	−4.23	Up
3268	P17918	Pcna	Proliferating cell nuclear antigen	28.8	4.77	−2.21	Up
3281	P12710	Fabp1	Fatty acid-binding protein	14.2	8.56	4.01	Down

^a^Spot No. generated by DeCyder image analysis software (v 7.0, GE Healthcare), referencing the spots shown on the representative image in Supplementary Fig. 5.

^b^Accession number from NCBI database.

^c^Gene symbol from NCBI database. All genes are *Mus musculus*.

^d^Average ratio of protein expression.

**Table 2:** Identification of picked proteins differentially expressed between KO villus and KO crypt.

Spot No.^a^	Accession No.^b^	Gene^c^	Protein Name	Protein MW (kDa)	Protein PI	Average fold change^d^	Overall trend
1118	P50544	Acadvl	Very long-chain specific acyl-CoA dehydrogenase, mitochondrial	70.8	8.75	2.61	Down
1266	P68373	Tuba1c	Tubulin alpha-1C chain	49.9	5.10	−2.15	Up
1320	Q3TEN9	Gyk	Glycerol kinase	57.4	5.76	−2.64	Up
1496	Q8VH52	Eif4a1	Eukaryotic translation initiation factor 4A1	16.1	5.15	2.78	Down
1732	Q9D154	Serpinb1a	Leukocyte elastase inhibitor A	42.5	6.21	−3.20	Up
1794	Q3TJ66	Aldob	Fructose-bisphosphate aldolase	39.5	8.27	−2.06	Up
1800	B2CY77	Rpsa	Laminin receptor	32.8	4.87	4.23	Down
1952	P14869	Rplp0	60S acidic ribosomal protein P0	34.2	6.25	−3.78	Up
2346	Q9Z1R9	Prss1	protease, serine 1 (trypsin 1)	26.1	4.94	4.23	Down
2529	Q3U0B3	Dhrs11	Dehydrogenase	28.3	6.34	−3.33	Up
3254	E9Q223	Hbb-bs	Protein Hbb-bs	11.1	6.37	18.87	Down
3261	P51162	Fabp6	Gastrotropin	14.5	6.15	−6.16	Up

^a^Spot No. generated by DeCyder image analysis software (v 7.0, GE Healthcare), referencing the spots shown on the representative image in Supplementary Fig. 5.

^b^Accession number from NCBI database.

^c^Gene symbol from NCBI database. All genes are *Mus musculus*.

^d^Average ratio of protein expression.

**Table 3:** Identification of picked proteins differentially expressed between WT villus and KO villus.

Spot No.^a^	Accession No.^b^	Gene^c^	Protein Name	Protein MW (kDa)	Protein PI	Average fold change^d^	Overall trend
1372	P56480	Atp5b	ATP synthase subunit beta, mitochondrial	56.3	5.34	−2.28	Up
1400	NP_032974	Psmc3	26S protease regulatory subunit 6A	44.6	5.02	−2.31	Up
1789	Q9D154	Serpinb1a	Leukocyte elastase inhibitor A	42.5	6.21	3.94	Down
1794	Q3TJ66	Aldob	Fructose-bisphosphate aldolase	39.5	8.27	2.84	Down
1870	Q3UAS2	Capza1	capping protein (actin filament) muscle Z-line, alpha 1	32.9	5.55	−2.32	Up
1875	Q3U804	Actb	Actin, Beta	41.8	6.30	−2.22	Up
2537	P10649	Gstm1	Glutathione S-transferase Mu 1	26.0	7.94	2.09	Down
2541	P35282	Rab21	Ras-related protein Rab-21	24.1	7.94	4.58	Down
2992	A0A087WQ08	Fam135a	Family with sequence similarity 135	30.5	7.01	2.4	Down
3254	E9Q223	Hbb-bs	Protein Hbb-bs	11.1	6.37	2.29	Down
3257	Q9Z1R9	Prss1	protease, serine 1 (trypsin 1)	26.1	4.94	2.14	Down
3281	P12710	Fabp1	Fatty acid-binding protein	14.2	8.56	2.16	Down

^a^Spot No. generated by DeCyder image analysis software (v 7.0, GE Healthcare), referencing the spots shown on the representative image in Supplementary Fig. 6.

^b^Accession number from NCBI database.

^c^Gene symbol from NCBI database. All genes are *Mus musculus*.

^d^Average ratio of protein expression.

**Table 4:** Identification of picked proteins differentially expressed between WT crypt and KO crypt.

Spot No.^a^	Accession No.^b^	Gene^c^	Protein Name	Protein MW (kDa)	Protein PI	Average fold change^d^	Overall trend
1875	Q3U804	Actb	Actin, Beta	41.8	6.30	−3.16	Up
2537	P10649	Gstm1	Glutathione S-transferase Mu 1	26.0	7.94	2.03	Down
3235	P51162	Fabp6	Gastrotropin	14.5	6.15	2.27	Down

^a^Spot No. generated by DeCyder image analysis software (v 7.0, GE Healthcare), referencing the spots shown on the representative image in Supplementary Fig. 6.

^b^Accession number from NCBI database.

^c^Gene symbol from NCBI database. All genes are *Mus musculus*.

^d^Average ratio of protein expression.

**Table 5:** Expression of the miRNA and its corresponding protein target.

Protein							miRNA				
Spot No.^a^	Protein Name	Gene^c^	Expression Level (Fold change)^b^				miRNA	Relative Expression Level			
			WTV	WTC	KOV	KOC		WTV	WTC	KOV	KOC
1320	Glycerol kinase	Gyk	1	2.58	1	2.64	miR-33-5p	1	0.20	1.76	0.34
1789	Leukocyte elastase inhibitor A	Serpinb1a	1	0.85	0.25	0.85	miR-221-3p	1	1.22	1.64	1.11
1800	Laminin receptor	Rpsa	2.58	1	4.23	1	miR-200c-5p	1	2.20	0.80	1.05
1875	Actin, beta	Actb	1	1	2.22	3.16	miR-212-3p	1	1.06	0.67	0.61

Abbreviations: WTV: Wild-type villus, WTC: Wild-type crypt, KOV: PepT1 KO villus, KOC: PepT1 KO crypt.

^a^Spot No. generated by DeCyder image analysis software (v 7.0, GE Healthcare), referencing the spots shown on the representative image in Supplementary Fig. 5, 6.

^b^Average ratio of protein expression.

^c^Gene symbol from NCBI database. All genes are *Mus musculus*.
