# Supplementary material for: PepT1 Expression Helps Maintain Intestinal Homeostasis by Mediating the Differential Expression of miRNAs along the Crypt-Villus Axis
Source: Sci Rep. 2016 Jun 1;6:27119. doi: 10.1038/srep27119 (PMC4890533; doi:10.1038/srep27119)
Supplement: Supplementary Information [file srep27119-s1.pdf]

PepT1 Expression Helps Maintain Intestinal Homeostasis by Mediating the Differential Expression of miRNAs along the Crypt-Villus Axis

Yuchen Zhang[1,*], Emilie Viennois[1], Mingzhen Zhang[1], Bo Xiao[1, 3], Moon Kwon Han[1], Lewins Walter[1], Pallavi Garg[4], Didier Merlin [1, 2]

[1]Center for Diagnostics and Therapeutics, Institute for Biomedical Sciences, Georgia State University, Atlanta, Georgia, 30302, USA

[2]Atlanta Veterans Affairs Medical Center, Decatur, Georgia, 30033, USA

[3]Institute for Clean Energy and Advanced Materials, Faculty for Materials and Energy, Southwest University, Chongqing, 400715, P. R. China

[4]Department of Biology, Georgia State University, Atlanta, Georgia, 30302, USA

[*]**Corresponding author:** Yuchen Zhang, Institute for Biomedical Sciences, Georgia State University, Atlanta, GA, Tel.: +1 (404) 413 5590; Fax: +1 (404) 413 3580; Email: yzhang42@gsu.edu

Supplementary Figure 1. Expression of PepT1 and Lgr5 in isolated epithelial cells of the jejunum from BL-6 WT mice. Total RNAs were extracted from the different fractions collected from BL-6 WT mice using the low-temperature method, and the expression levels of PepT1 (as a villus marker) and Lgr5 (as a crypt marker) were assessed by qRT-PCR.

Supplementary Figure 2. Expression of Muc2, Lgr5, and mPepT1 in WT and KO mice. The expression levels of (*a*) Muc2, (*b*) Lgr5, and (*c*) mPepT1 were assessed by immunofluorescence. Muc2, Lgr5 and mPepT1 were immunostained using anti-Muc2, anti-Lgr5, and anti-PepT1, respectively (FITC, green). F-actin was stained using phalloidin (TRITC, red), and cell nuclei were stained using DAPI (blue). Separate pictures were taken at 20× for each filter, and the images were merged. Scale bar, 50 µm.

Supplementary Figure 3. Clustering graph of selected miRNAs. MicroRNA microarray results demonstrated that different miRNA profiles were ovserved in crypts and villi of WT and PepT1 KO mice. We selected miRNAs that exhibited *P* value <0.05 and signal strengths > 500.

Supplementary Figure 4. Expression levels of selected miRNAs in crypt and villus epithelial cells from BL-6 WT and PepT1 KO mice. The expression levels of 36 selected miRNAs were verified by qRT-PCR (n= 5/group; * $P< 0.05$, ** $P< 0.005$, and *** $P< 0.001$).

Supplementary Figure 5. PepT1 expression disturbs normal differential protein expression along the crypt-villus axis. 2D-DIGE DeCyder BVA (Biological Variation Analysis) showing a representative gel image with labeled sample of difference. Changes in

protein expression were compared between villi and crypts, with a pooled internal standard included. The master gel image shows the locations of the differentially expressed proteins in each comparison. *a.* 43 spots with > 2.0-fold differences in WT villi vs. WT crypts. *b.* 72 spots with > 2.0-fold differences in KO villi vs. KO crypts.

Supplementary Figure 6. PepT1 expression altered the normal protein profile in villus and crypt, respectively. 2D-DIGE DeCyder BVA (Biological Variation Analysis) showing a representative gel image with labeled sample of difference. Changes in protein expression were compared between BL-6 WT and PePT1 KO mice, with a pooled internal standard included. The master gel image shows the locations of the differentially expressed proteins in each comparison. *a.* 24 spots with > 2.0-fold differences in WT villi vs. KO villi. *b.* 5 spots with > 2.0-fold differences in WT crypts vs. KO crypts.

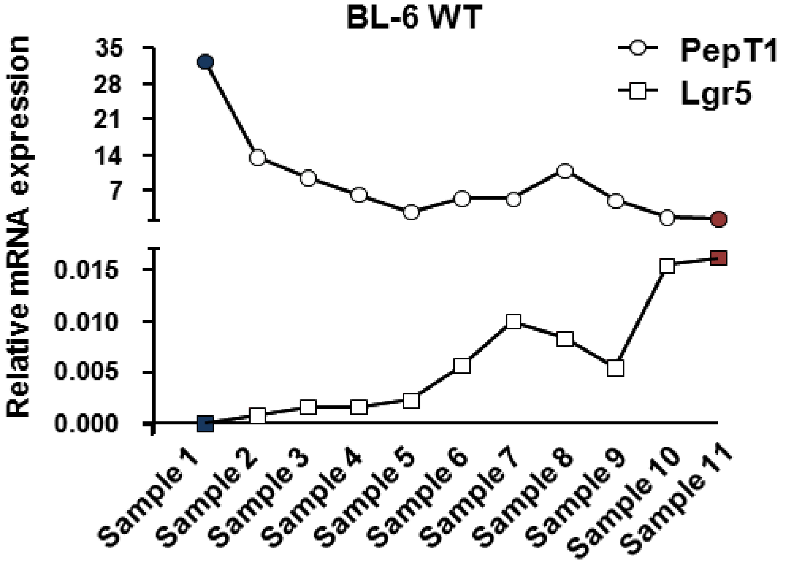

Supplementary Figure 1

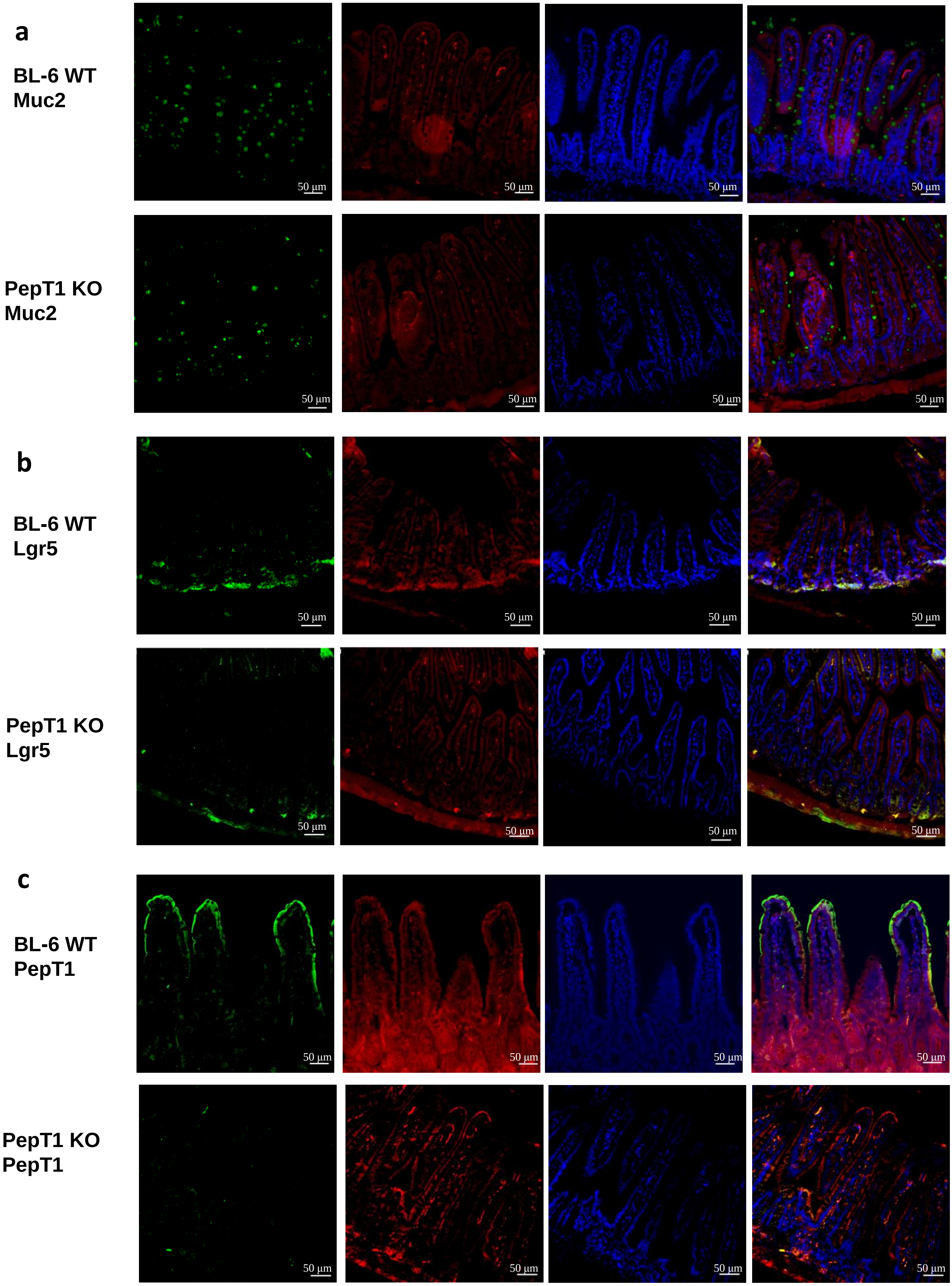

Supplementary Figure 2

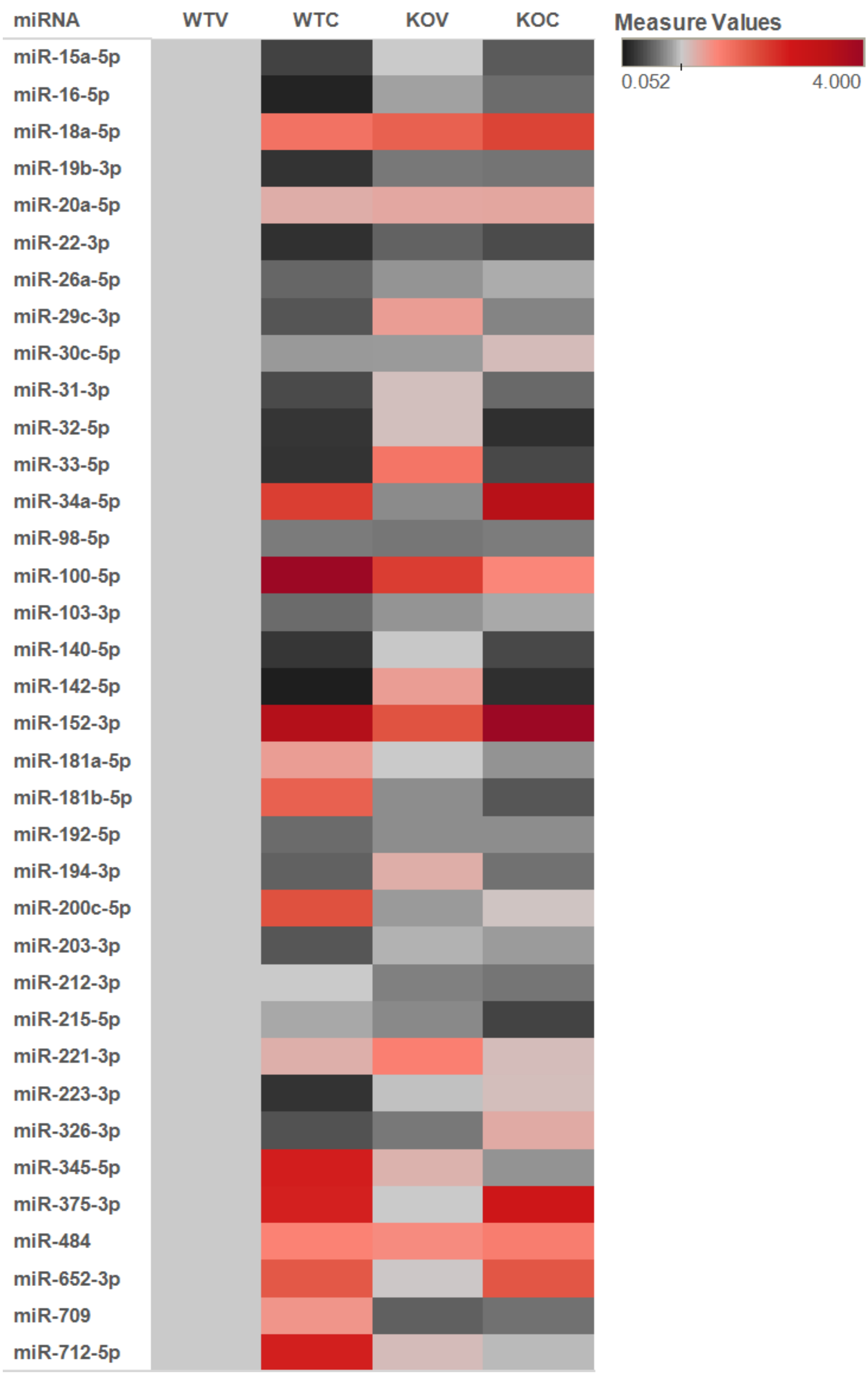

Supplementary Figure 3

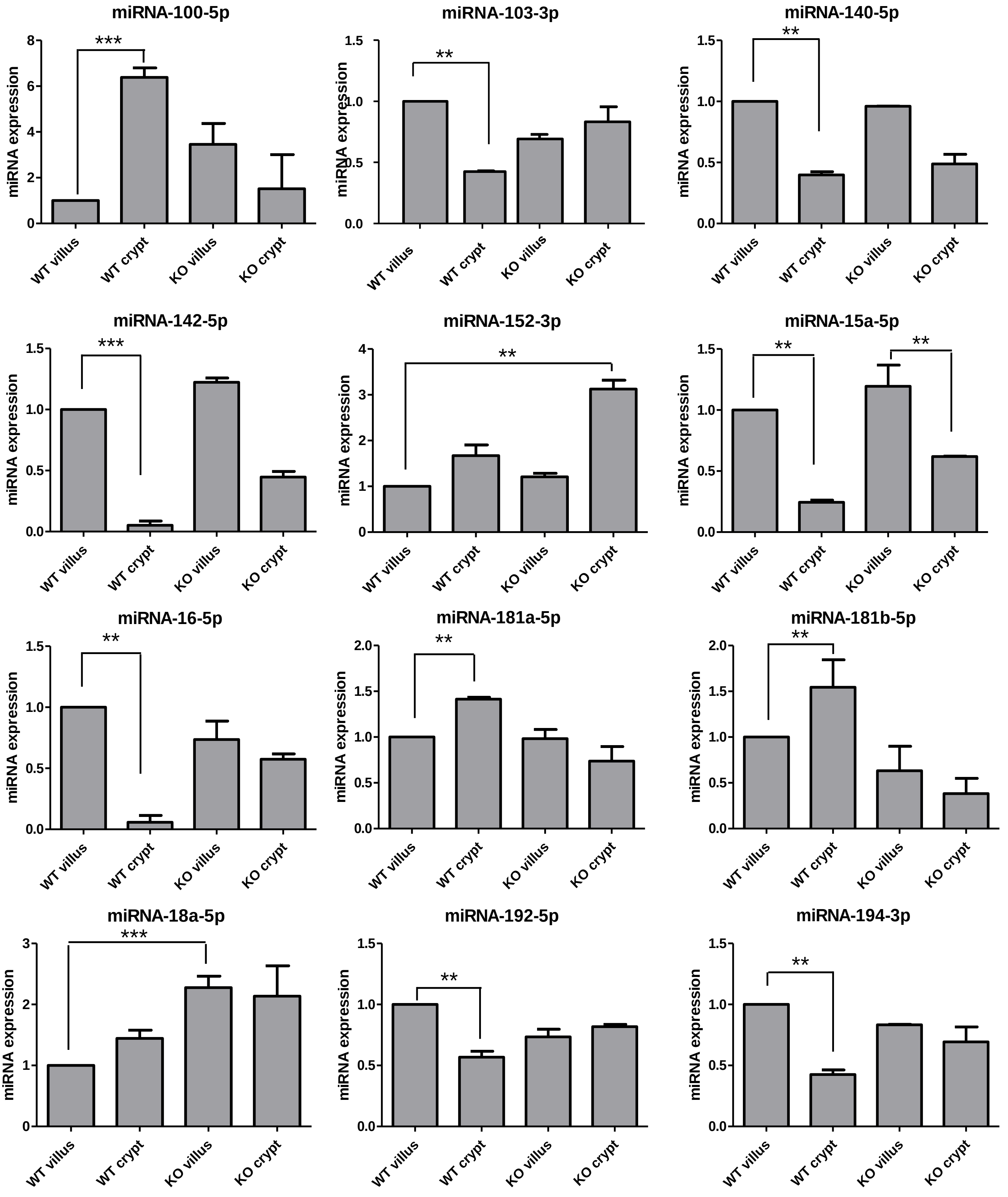

Supplementary Figure 4

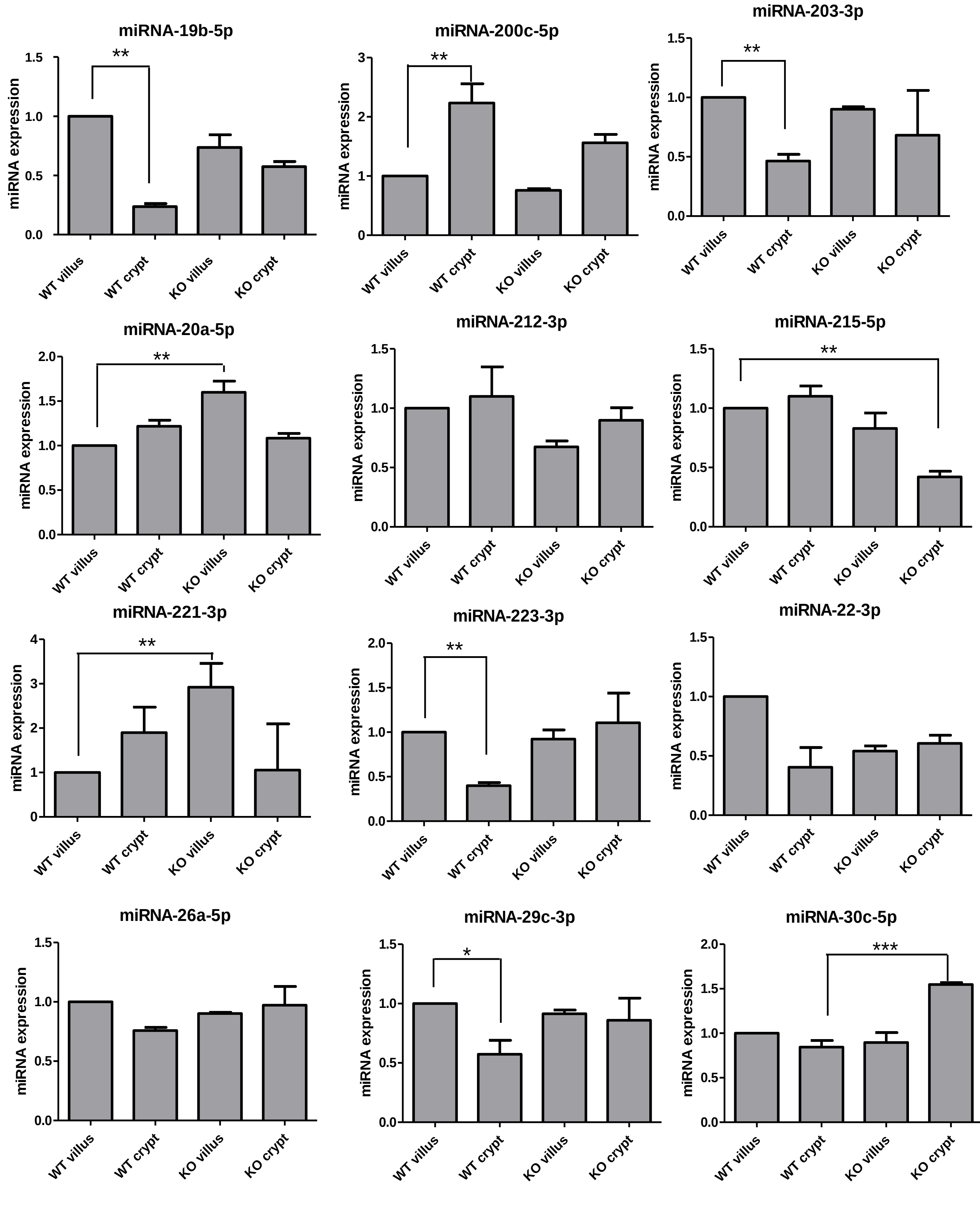

Supplementary Figure 4

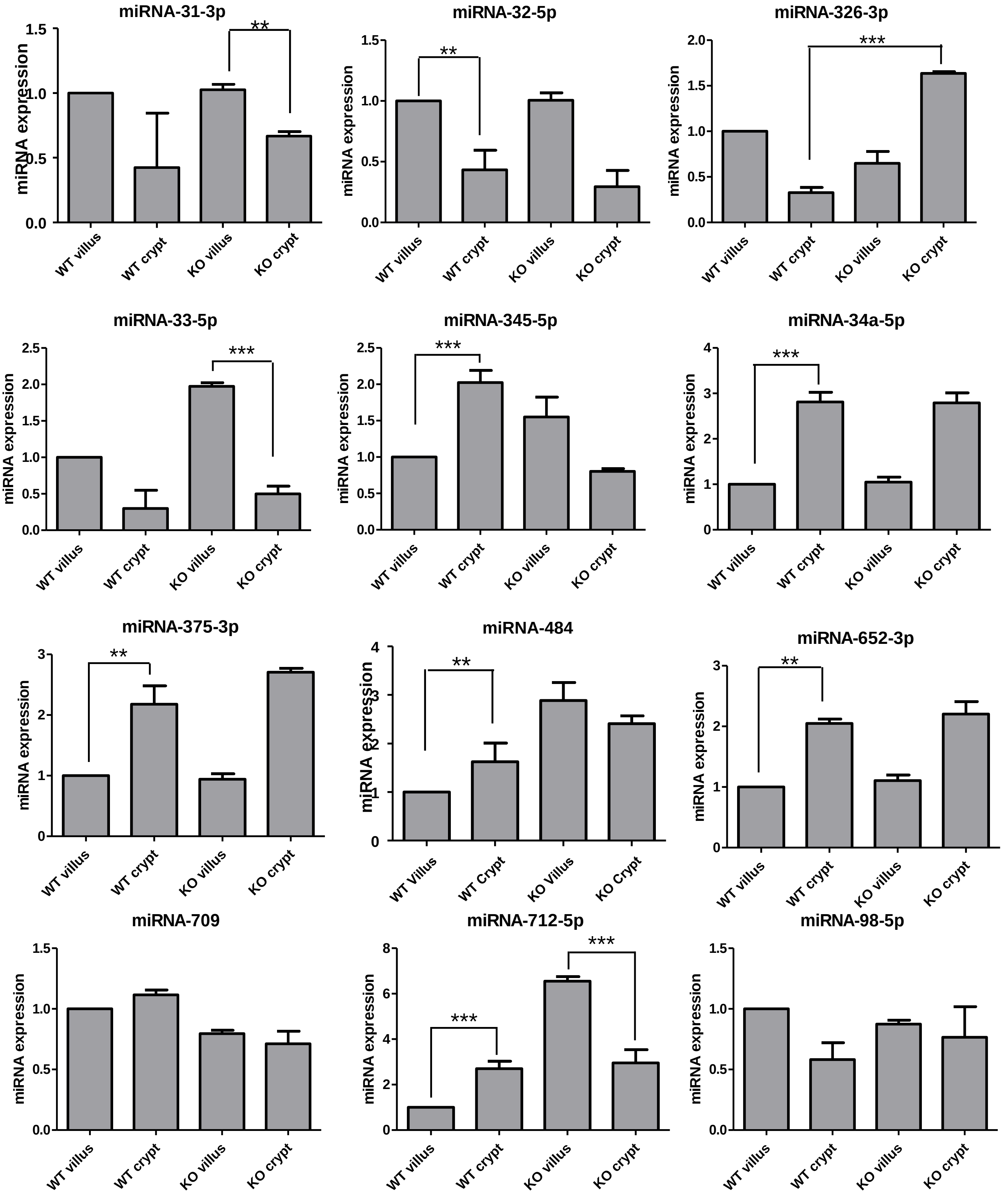

Supplementary Figure 4

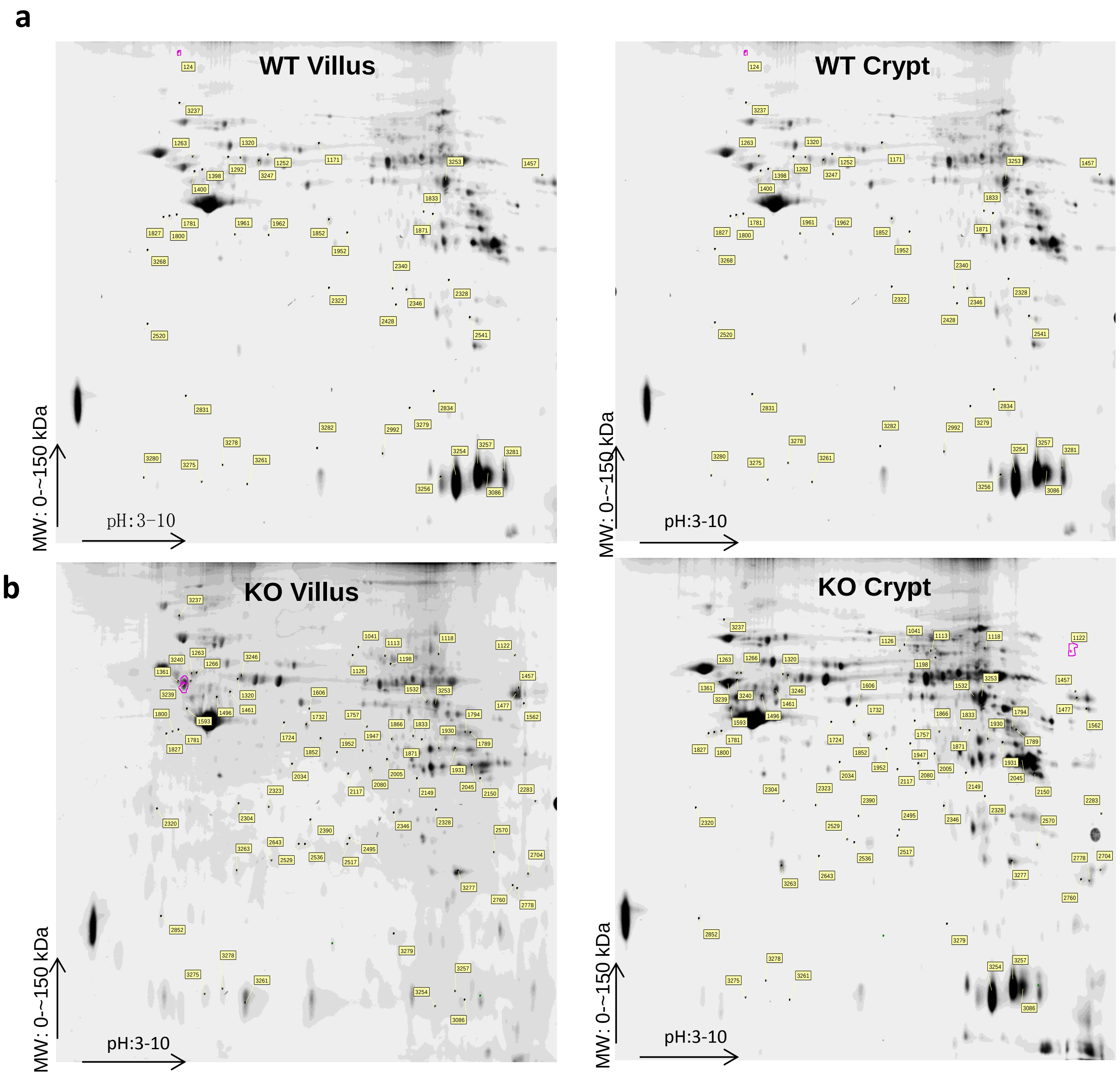

Supplementary Figure 5

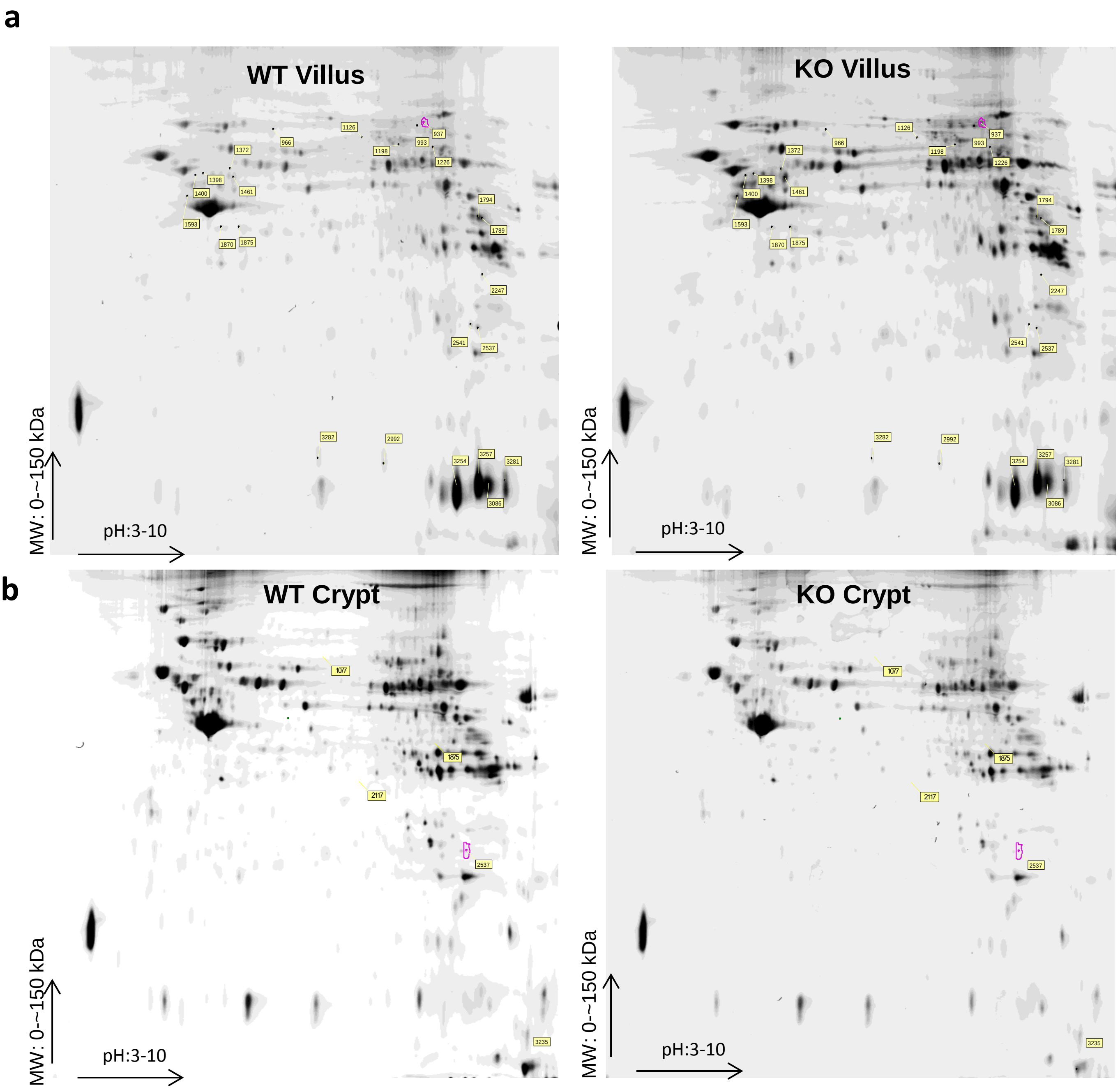

Supplementary Figure 6

Supplementary Table 1. List of primers used in this study

Primer	Sequences
36B4 F	TCCAGGCTTTGGGCATCA
36B4 R	CTTTATCAGCTGCACATCACTCAGA
mPepT1 F	CTCCATCTTCTACCTGTCCATCAACGCA
mPepT1 R	GCTACGGTTCCTGAAGCGGTTTTTGACT
mmu-miR-142-5p F	CATAAAGTAGAAAGCACTACT
mmu-miR-16-5p F	TAGCAGCACGTAAATATTGGCG
mmu-miR-22-3p F	AAGCTGCCAGTTGAAGAACTGT
mmu-miR-194-3p F	CCAGTGGGGCTGCTGTTATCTG
mmu-miR-33-5p F	GTGCATTGTAGTTGCATTGCA
mmu-miR-223-3p F	TGTCAGTTTGTCAAATACCCCA
mmu-miR-32-5p F	TATTGCACATTACTAAGTTGCA
mmu-miR-140-5p F	CAGTGGTTTTACCCTATGGTAG
mmu-miR-15a-5p F	TAGCAGCACATAATGGTTTGTG
mmu-miR-31-3p F	TGCTATGCCAACATATTGCCATC
mmu-miR-326-3p F	CCTCTGGGCCCTTCCTCCAGT
mmu-miR-29c-3p F	TAGCACCATTTGAAATCGGTTA

mmu-miR-203-3p F	GTGAAATGTTTAGGACCACTAG
mmu-miR-19b-5p F	AGTTTTGCAGGTTTGCATCCAGC
mmu-miR-26a-5p F	TTCAAGTAATCCAGGATAGGCT
mmu-miR-103-3p F	AGCAGCATTGTACAGGGCTATGA
mmu-miR-192-5p F	CTGACCTATGAATTGACAGCC
mmu-miR-98-5p F	TGAGGTAGTAAGTTGTATTGTT
mmu-miR-30c-5p F	TGTAAACATCCTACACTCTCAGC
mmu-miR-215-5p F	ATGACCTATGATTTGACAGAC
mmu-miR-212-3p F	TAACAGTCTCCAGTCACGGCCA
mmu-miR-221-3p F	AGCTACATTGTCTGCTGGGTTTC
mmu-miR-20a-5p F	TAAAGTGCTTATAGTGCAGGTAG
mmu-miR-181a-5p F	AACATTCAACGCTGTCGGTGAGT
mmu-miR-709 F	GGAGGCAGAGGCAGGAGGA
mmu-miR-484 F	TCAGGCTCAGTCCCCTCCCGAT
mmu-miR-18a-5p F	TAAGGTGCATCTAGTGCAGATAG
mmu-miR-181b-5p F	AACATTCATTGCTGTCGGTGGGT
mmu-miR-652-3p F	AATGGCGCCACTAGGGTTGTG
mmu-miR-34a-5p F	TGGCAGTGTCTTAGCTGGTTGT

mmu-miR-200c-5p F	CGTCTTACCCAGCAGTGTTTGG
mmu-miR-375-3p F	TTTGTTCGTTCGGCTCGCGTGA
mmu-miR-712-5p F	CTCCTTCACCCGGGCGGTACC
mmu-miR-345-5p F	GCTGACCCCTAGTCCAGTGCTT
mmu-miR-152-3p F	TCAGTGCATGACAGAACTTGG
mmu-miR-100-5p F	AACCCGTAGATCCGAACTTGTG
Muc2 F	GACCTGACAATGTGCCCAGA
Muc2 R	GGCAAACACAGTCCTTGCAG
Lgr5 F	CTGCCCATCACACTGTCACT
Lgr5 R	GCAGAGGCGATGTAGGAGAC
